# Hypothalamic thermoregulatory neurons divergently modulate isoflurane anesthesia via temperature dependent and independent mechanisms

**DOI:** 10.1016/j.isci.2026.115542

**Published:** 2026-03-30

**Authors:** Shuang Cai, Wen Z. Yang, Mao Xu, Huimin Chen, Liang Zhou, Tianyuan Luo, Shouyang Yu, Kan Zhang, Haiying Wang, Tian Yu, Jijian Zheng, Wei L. Shen

**Affiliations:** 1 Department of Anesthesiology, Shanghai Children’s Medical Centre, Shanghai Jiao Tong University, Shanghai 200127, China; 2 Key Laboratory of Brain Science, Key Laboratory of Anesthesia and Organ Protection of Ministry of Education (In Cultivation), Zunyi Medical University, Zunyi, Guizhou 563006, China; 3 Department of Anesthesiology, Affiliated Hospital of Zunyi Medical University, Zunyi, Guizhou 563000, China; 4 School of Life Science and Technology, ShanghaiTech University, Shanghai 201210, China; 5 Zunyi Medical University, Zunyi, Guizhou 563006, China; 6 Department of Anesthesiology, Nanfang Hospital, Southern Medical University, Guangzhou, Guangdong 510515, China

**Keywords:** health sciences, human physiology, neuroscience

## Abstract

Perioperative hypothermia is common clinically. The neural mechanisms underlying general anesthesia and the hypothermia it induces remain elusive. We found that lower core temperature (T_core_) deepened anesthesia and delayed emergence, which showed a logarithmic relationship below 31.9°C. Chemogenetic activation of brain-derived neurotrophic factor in the lateral preoptic area (LPO^BDNF^) neurons before isoflurane administration lowered T_core_ and increased isoflurane induced burst-suppression ratio (BSR), δ power, and recovery time, while its optogenetic activation during isoflurane did not change T_core_ and anesthesia depth. Conversely, chemogenetic activation of dorsomedial hypothalamus glutamatergic (DMH^Vglut2^) neurons increased T_core_, attenuated anesthesia depth and promoted recovery. Stage-specific optogenetic activation of DMH^Vglut2^ neurons and its downstream raphe pallidus (RPa) projection during isoflurane exposure induced cortical activation without T_core_ alteration. Our study indicates that LPO^BDNF^ neurons modulate isoflurane anesthesia primarily through temperature-dependent mechanism, while DMH^Vglut2^ neurons and the DMH^Vglut2^ to RPa pathway have a temperature-independent function in modulating isoflurane anesthesia.

## Highlights

• Anesthetic emergence delays logarithmically as T_core_ falls below 31.9°C in mice
• LPO^BDNF^ neurons potentiates anesthetic depth via a temperature-dependent manner
• The DMH^Vglut2^→RPa pathway accelerates emergence by temperature-independent mechanism

## Graphical abstract

**Figure:**
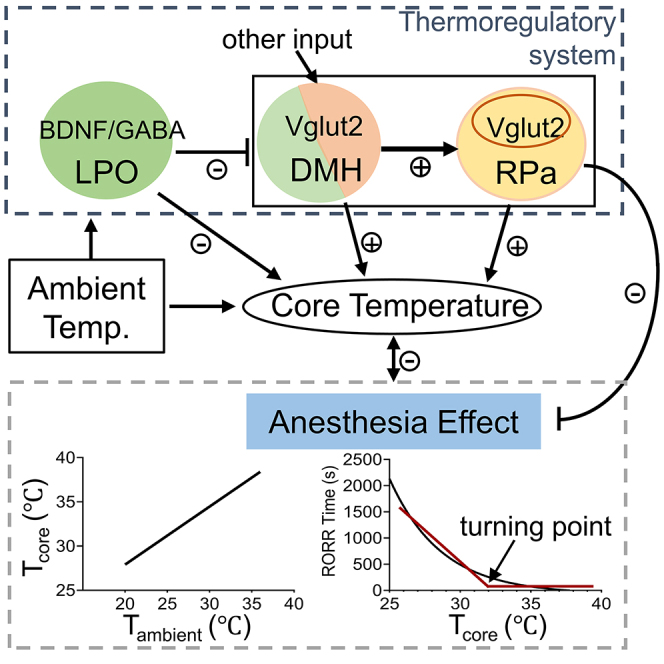

## Introduction

General anesthesia induces a state of unconsciousness often accompanied by suppression of multiple physiological functions, including thermoregulation, which frequently leads to hypothermia, respiratory inhibition, and circulatory inhibition.^1^^,^^2^ Clinical studies report that approximately 70% of surgical patients develop intraoperative mild hypothermia with core temperature (T_core_) less than 36.0°C,^3^^,^^4^ which increases the risk of surgical complications, including coagulopathy, infection, shivering, and delayed recovery.^5^^,^^6^ Recent studies reported that specific neural circuits that regulate sleep-wakefulness are involved in general anesthetics-induced unconsciousness and emergence,^7^^,^^8^^,^^9^ and also share a significant overlap with thermoregulatory circuits.^10^^,^^11^^,^^12^ These lead us to propose that the thermoregulatory neurons may also be involved in modulating the general anesthesia state, while the mechanisms remain elusive.

T_core_ is actively and tightly controlled by hypothalamic neurocircuits under physiological conditions. The hypothalamic preoptic area (POA) is a well-established center for thermoregulation.^13^^,^^14^^,^^15^ Distinct regions and neuronal populations within the POA contribute to different aspects of thermoregulation.^16^ Activation of γ-aminobutyric acid (GABAergic) neurons, mostly with BDNF expressing in the lateral preoptic area (LPO) induces torpor-like profound hypothermia without cold defense responses.^15^ The dorsomedial hypothalamus glutamatergic (DMH) constitutes a principal efferent pathway of the LPO.^15^^,^^16^ In contrast to the LPO, DMH is responsible for increasing T_core_ in responding to cold.^17^^,^^18^ The DMH contains both glutamatergic and GABAergic neurons.^19^^,^^20^ Chemogenetic activation of dorsomedial hypothalamus glutamatergic (DMH^Vglut2^) neurons induces hyperthermia via their raphe pallidus (RPa) projections.^21^ Beyond thermoregulation, the DMH promotes wakefulness via projecting to several key nuclei, including dorsal raphe nucleus (DR), ventral tegmental area (VTA), and locus coeruleus (LC).^22^^,^^23^ Although the function of DMH^GABA^ neurons in sevoflurane emergence has been implicated,^24^ overall, how thermoregulatory DMH neurons contribute to anesthesia need deeper exploration. Thus, we hypothesized that brain-derived neurotrophic factor in the lateral preoptic area (LPO^BDNF^) and DMH^Vglut2^ neurons play a substantial role of isoflurane-induced anesthesia.

In this study, we find that low T_core_ (less than 31.9°C) increased the depth of isoflurane anesthesia and delayed recovery. Chemogenetic activation of LPO^BDNF^ neurons before isoflurane administration lowered T_core_ and increased isoflurane induced electroencephalogram (EEG) burst-suppression ratio (BSR), δ power during anesthesia, and prolonged recovery time, while its optogenetic activation during isoflurane exposure did not change T_core_ and anesthesia depth. Conversely, chemogenetic activation of DMH^Vglut2^ neurons increased T_core_, attenuated anesthesia depth during anesthesia and promoted recovery. Optogenetic activation of DMH^Vglut2^ neurons and its downstream RPa projection during anesthesia attenuated anesthesia depth without T_core_ alteration, suggesting the existence of temperature-independent mechanism for direct modulation of anesthesia.

## Results

### Core temperature and anesthesia effect changes along with ambient temperature under isoflurane

**Figure 1:**
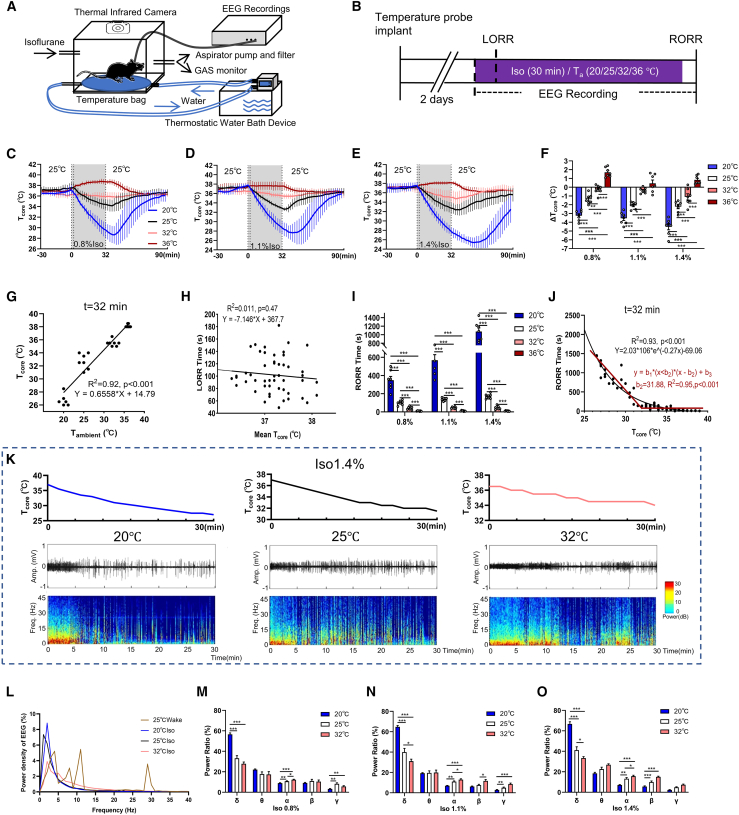
T_core_ negatively affects anesthesia depth in mice (A) Schematic diagram depicting the T_a_-controlling device for isoflurane-induced anesthesia. (B) Schematic of protocol for the isoflurane anesthesia procedure, with simultaneous T_core_ monitoring and EEG recordings. (C–E) T_core_ changes in response to different T_a_ exposures (20°C/25°C/32°C/36°C) under 0.8% (C), 1.1% (D), and 1.4% (E) isoflurane, respectively. Gray shading indicates isoflurane anesthesia exposure (starting at *t* = 0, and ceasing at *t* = 32 min. LORR time is ∼2 min). (F) Changes in T_core_ (measured at *t* = 32 min) under different isoflurane concentrations at different T_a_. (25°C T_a_: 0.8%, −1.36°C ± 0.21°C, 1.1%, −2.08°C ± 0.15°C, 1.4%, −2.22°C ± 0.23°C; 32°C T_a_: 0.8%, −0.27°C ± 0.19°C, 1.1%, −0.38°C ± 0.12°C, 1.4%, −1.11°C ± 0.36°C, 36°C T_a_: 0.8%, 1.70°C ± 0.20°C, 1.1%, 0.44°C ± 0.38°C, 1.4%, 0.84°C ± 0.18°C; 0.8%, 20°C vs. 25°C, *p* < 0.001, 32°C vs. 25°C, *p* < 0.001, 36°C vs. 25°C, *p* < 0.001; 1.1%, 20°C vs. 25°C, *p* < 0.001, 32°C vs. 25°C, *p* < 0.001, 36°C vs. 25°C, *p* < 0.001; 1.4%, 20°C vs. 25°C, *p* < 0.001, 32°C vs. 25°C, *p* < 0.001, 36°C vs. 25°C, *p* < 0.001; *n* = 7). (G) Linear regression model of T_a_ and T_core_ of mice at the time of isoflurane ceasing (*t* = 32 min), Y = 0.66∗X + 14.79, *R*^2^ = 0.92, *p* < 0.001. (H) LORR time plotted against T_core_ at the start of isoflurane (*t* = 0 min) for all tested mice as shown in (I). No linear relationship between LORR time and T_core_ was detected (*R*^2^ = 0.01, *p* = 0.47). (I and J) Statistical analysis of RORR time across all groups. (25°C T_a_: 0.8%, 99.71 ± 25.45 s, 1.1%, 140.14 ± 16.36 s, 1.4%, 171.57 ± 27.08 s; 32°C T_a_: 0.8%, 39.43 ± 16.21 s, 1.1%, 46.00 ± 8.62 s, 1.4%, 45.71 ± 18.47 s; 0.8%, 20°C vs. 25°C, *p* < 0.001, 32°C vs. 25°C, *p* < 0.001, 36°C vs. 25°C, *p* < 0.001; 1.1%, 20°C vs. 25°C, *p* < 0.001, 32°C vs. 25°C, *p* < 0.001, 36°C vs. 25°C, *p* < 0.001; 1.4%, 20°C vs. 25°C, *p* < 0.001, 32°C vs. 25°C, *p* < 0.001, 36°C vs. 25°C, *p* < 0.001; *n* = 7). (J) Logarithmic regression model (one-phase decay) of RORR time as a function of T_core_ at the time of isoflurane cessation (*t* = 32 min) for all tested mice, Y = 20.3∗10^5^∗eˆ(−0.27∗x) −69.06, *R*^2^ = 0.93, *p* < 0.001. Inflection point (31.88°C) was calculated by nonlinear regression model: *y* = b_1_ ∗(*x* < b_2_) ∗ (*x* − b_2_) + b_3_). (b_1_ = −307.47, b_2_ = 31.88, b_3_ = 60.48). (K) Representative T_core_ (top), raw EEG traces (middle), and EEG power spectra (bottom) at different T_a_ under 1.4% isoflurane. (L) Raw EEG power density of mice in wake and 1.4% isoflurane at different T_a_ (30 min). (M–O) EEG power percentage of different frequency bands during 0.8% (M; δ band, 20°C vs. 25°C, F (4, 130) = 153.02, *p* < 0.001, 32°C vs. 25°C, F (4, 130) = 4.96, *p* = 0.13; γ band, 20°C vs. 25°C, F (4, 130) = 6.92, *p* = 0.047; *n* = 7), 1.1% (N; δ band: 20°C vs. 25°C, F (4, 130) = 142.32, *p* < 0.001, 32°C vs. 25°C, F (4, 130) = 12.40, *p* = 0.030; *n* = 7), and 1.4% (O; δ band: 20°C vs. 25°C, F (4, 130) = 139.71, *p* < 0.001, 32°C vs. 25°C, F (4, 130) = 13.88, *p* = 0.0014; *n* = 7; α band, 20°C vs. 25°C, F (4, 130) = 8.30, *p* = 0.023; *n* = 7) isoflurane anesthesia procedures, respectively. LORR, the loss of righting reflex; RORR, the recovery of righting reflex; EEG, electroencephalogram. All data are presented as mean ± SEM, ∗*p* < 0.05, ∗∗*p* < 0.01, and ∗∗∗*p* < 0.001, n.s., not significant (F) and (I) were analyzed by multiple *t* test, (G) and (H) were analyzed by linear regression analysis, (J) was analyzed by non-linear regression (one-phase decay) analysis, and (M–O) were analyzed by two-way RM ANOVA followed by Bonferroni’s multiple comparisons test.

Under the various ambient temperatures (T_a_) at 20°C, 25°C, 32°C, and 36°C (Figures 1A and 1B), T_core_ was significantly changed under isoflurane exposure. Specifically, at 20°C, 0.8%, 1.1%, and 1.4% isoflurane reduced T_core_ by 3.24°C ± 0.58°C, 3.54°C ± 0.69°C, and 4.51°C ± 0.93°C, respectively (*n* = 7; Figures 1C–1F). The same pattern changes were noted in other concentrations and ambient temperature (Figures 1C–1F). Quantitative analysis showed a linear relationship between T_a_ and T_core_ (*R*^2^ = 0.92; Figure 1G).

During anesthesia induction, T_core_ remained stable (36°C–37°C) under various T_a_ and showed no correlation with loss of righting reflex (LORR) time (*R*^2^ = 0.01, *p* = 0.47; Figure 1H). However, recovery of righting reflex (RORR) time was inversely correlated with T_core_ measured at the cessation of anesthesia (*t* = 32 min; Figure 1I). RORR time under 0.8%, 1.1%, and 1.4% isoflurane at 20°C T_a_ were 348.57 ± 107.46 s, 568.57 ± 159.21 s, and 1074.29 ± 227.44 s, respectively, while at 36°C T_a_, they were only 9.43 ± 2.88 s, 10.57 ± 4.61 s, and 9.71 ± 3.64 s, respectively (*n* = 7; Figure 1I). Quantitative analysis showed a strong logarithmic relationship between RORR time and T_core_ (*R*^2^ = 0.93, *p* < 0.001), and the turning point was identified at ∼31.88°C, beyond which further increase of T_core_ had minimal effect on anesthesia emergence (Figure 1J).

EEG spectral power distributions varied significantly with T_a_ during isoflurane anesthesia (Figures 1K and 1L). Power spectral analysis further supported the negative impact of T_core_ on anesthetic depth. Delta (δ) power (1–4 Hz) increased at lower T_a_ (20°C) and reduced at higher T_a_ (32°C). Under 0.8% isoflurane, δ power values were 56.66% ± 5.14% at 20°C T_a_, 33.16% ± 9.64% at 25°C T_a_, and 27.81% ± 6.46% at 32°C T_a_ (*n* = 7). The difference showed between 20°C and 25°C (*p* < 0.001), while there was no difference between 32°C and 25°C (*p* = 0.096; Figure 1M). Under 1.1% and 1.4% isoflurane, δ power significantly varied with ambient temperature changes (Figures 1N and 1O).

### Chemogenetic activation of LPO^BDNF^ neurons deepens anesthesia by decreasing T_core_

**Figure 2:**
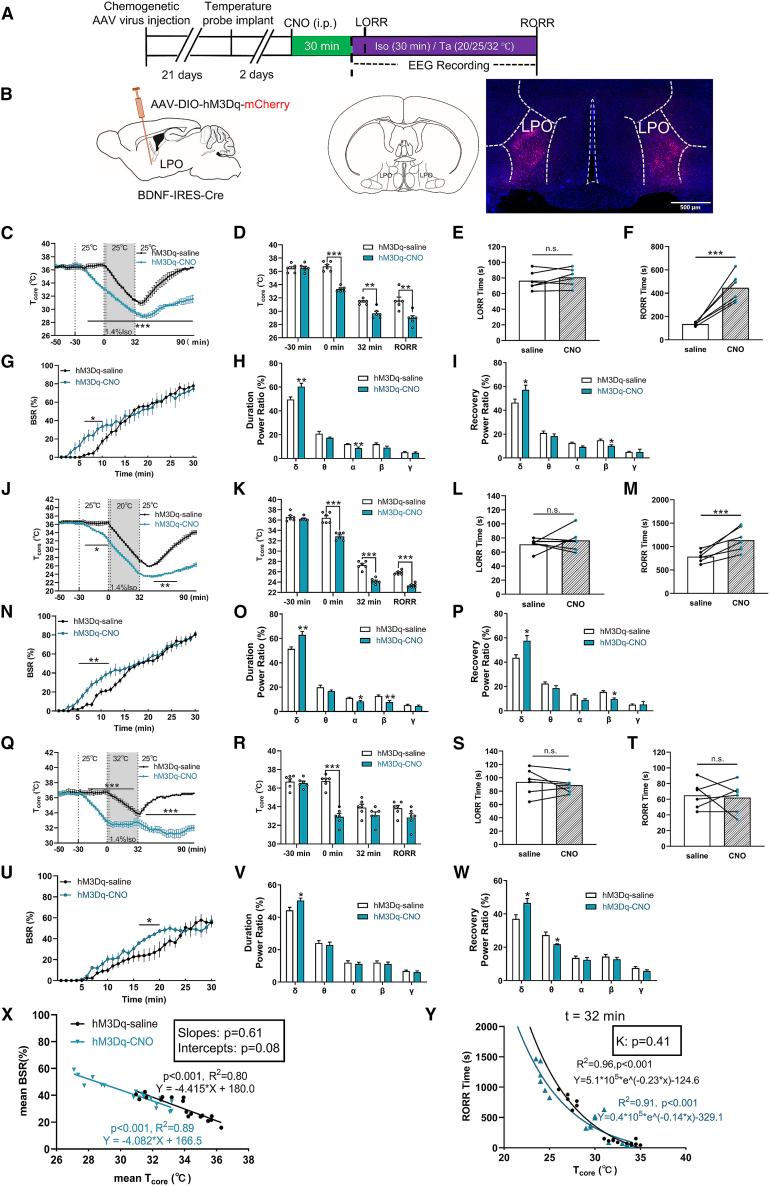
Chemogenetic activation of LPO^BDNF^ neurons deepens isoflurane-induced anesthesia by decreasing T_core_ (A) Protocol for chemogenetic experiments, CNO was delivered 30 min prior to isoflurane-induced anesthesia. T_core_ and EEG were monitored during the anesthesia procedure. (B) Schematic representation of *AAV-DIO-hM3Dq-mCherry (hM3Dq)* injection into the LPO region of *BDNF-ires-Cre* mice (left). Representative image showing hM3Dq virus expressing in LPO region of mice (scale bars, 500 μm) (right). (C) T_core_ changes at T_a_ of 25°C under isoflurane in hM3Dq-saline and hM3Dq-CNO mice. T_core_ was recorded every 2 min (measured by intraperitoneal implantation). CNO injected at −30 min, isoflurane started at *t* = 0 min, induced LORR of mice within 2 min, and maintained for 30 min, isoflurane ceased at *t* = 32 min. (D) The values of T_core_ at different time under isoflurane in hM3Dq-saline and hM3Dq-CNO mice. T_core_ at *t* = −30 min, the time of isoflurane starting (*t* = 0 min), isoflurane cessation (*t* = 32 min) and at the RORR moment (t_RORR_). (E) Chemogenetic activation of LPO^BDNF^ neurons at T_a_ of 25°C did not influence LORR time. (F) Chemogenetic activation of LPO^BDNF^ neurons at T_a_ of 25°C prolonged the RORR time. (G) Burst suppression ratio (BSR) changes during isoflurane anesthesia procedure (30 min) in hM3Dq-saline and hM3Dq-CNO mice. BSR is plotted at every minute. (H) LPO^BDNF^ neural activation during isoflurane procedure (0–30 min) at T_a_ of 25°C displayed significant increase in δ power band (F (4, 50) = 26.59; *p* < 0.001; *n* = 6) analyzed from EEG recordings. (I) LPO^BDNF^ neural activation in recovery procedure (from isoflurane ceasing to RORR) at T_a_ of 25°C displayed increase in δ power band (F (4, 50) = 14.89; *p* = 0.046; *n* = 6) analyzed from EEG recordings. (J) T_core_ changes at T_a_ of 20°C under isoflurane in hM3Dq-saline and hM3Dq-CNO mice. (K) The values of T_core_ at different time under isoflurane in hM3Dq-saline and hM3Dq-CNO mice. (L) Chemogenetic activation of LPO^BDNF^ neurons at T_a_ of 20°C did not influence the LORR time. (M) Chemogenetic activation of LPO^BDNF^ neurons at T_a_ of 20°C prolonged the RORR time. (N) Burst suppression ratio (BSR) changes during isoflurane anesthesia procedure (30 min) in hM3Dq-saline and hM3Dq-CNO mice. (O) LPO^BDNF^ neural activation during isoflurane procedure (0–30 min) at T_a_ of 20°C displayed significant increase in δ power band (F (4, 50) = 33.76; *p* = 0.0035; *n* = 6). (P) LPO^BDNF^ neural activation in recovery procedure (from isoflurane ceasing to RORR) at T_a_ of 20°C displayed decrease in β power band (F (4, 50) = 25.72; *p* < 0.001; *n* = 6). (Q) T_core_ changes during isoflurane at T_a_ of 32°C in hM3Dq-saline and hM3Dq-CNO mice. (R) The values of T_core_ at different time under isoflurane in hM3Dq-saline and hM3Dq-CNO mice. (S and T) Chemogenetic activation of LPO^BDNF^ neurons at T_a_ of 32°C has no changes in LORR time (S) and RORR time (T). (U) BSR changes during isoflurane anesthesia procedure (0–30 min) at T_a_ of 32°C in hM3Dq-saline and hM3Dq-CNO mice. (V) LPO^BDNF^ neurons activation during isoflurane (0–30 min) at T_a_ of 32°C displayed significant increase in δ power band (F (4, 50) = 11.38; *p* = 0.0021; *n* = 6). (W) LPO^BDNF^ neural activation during recovery procedure (from isoflurane ceasing to RORR) at T_a_ of 32°C displayed significant increase in δ power band and decrease in θ power band. (X) The linear regression model of mean BSR and mean T_core_ of hM3Dq-saline and hM3Dq-CNO mice during isoflurane procedure (30 min). hM3Dq-saline, Y = −4.415∗X + 180.0, *R*^2^ = 0.80, *p* < 0.001; hM3Dq-CNO, Y = −4.082∗X + 166.5, *R*^2^ = 0.89, *p* < 0.001. Comparation within groups: slopes, *p* = 0.61; intercepts, *p* = 0.08. (Y) Logarithmic regression model (one-phase decay) of RORR time as a function of T_core_ at the time of isoflurane ceasing (*t* = 32 min) in hM3Dq-saline and hM3Dq-CNO groups. hM3Dq-saline, Y = 5.1∗10^5^∗eˆ(−0.23∗x)−124.6, *R*^2^ = 0.96, *p* < 0.001, hM3Dq-CNO, Y = 0.4∗10^5^∗eˆ(−0.14∗x) −329.1, *R*^2^ = 0.91, *p* < 0.001. *k* value within groups comparation showed no differences (*p* = 0.41). All data are presented as mean ± SEM, ∗*p* < 0.05, ∗∗*p* < 0.01, and ∗∗∗*p* < 0.001, ns, not significant, (D), (K), and (R) were analyzed by multiple *t* test, (E), (F), (L), (M), (S), and (T) were analyzed by paired *t* test, (G–I), (N–P), and (U–W) were analyzed by two-way RM ANOVA followed by Bonferroni’s multiple comparisons test, (X) was analyzed by linear regression analysis, and (Y) was analyzed by non-linear regression (one-phase decay) analysis.

Clozapine-N-oxide (CNO) administration (1 mg/kg, intraperitoneal [i.p.], Figure S1A) to activate LPO^BDNF^ prior to isoflurane exposure significantly reduced T_core_, and this hypothermic effect persisted throughout isoflurane exposure (Figures 2A–2D). While their activation did not affect LORR time (Figure 2E), it significantly prolonged anesthesia recovery time at 25°C T_a_ (hM3Dq-CNO 446.00 ± 121.86 s vs. hM3Dq-saline 135.67 ± 15.15 s; *n* = 6, *p* < 0.001; Figure 2F). Furthermore, chemogenetic activation significantly increased the burst suppression ratio (BSR) during anesthesia (Figure 2G). Power spectral analysis showed increased δ power (hM3Dq-CNO 55.34 ± 4.40% vs. hM3Dq-saline 44.59 ± 1.91%; *p* = 0.0091; Figure 2H) during anesthetic maintenance, as well as elevated δ power (CNO 57.64% ± 4.16% vs. saline 43.64% ± 2.46%; *p* = 0.046; Figure 2I) during recovery procedure at 25°C T_a_ (Figure S1B). The same pattern changes were noted in the LPO^BDNF^ activation mice at 20°C T_a_. Further T_core_ reduction (Figures 2J and 2K) had no difference of LORR (Figure 2L) but prolonged RORR (Figure 2M), increased BSR (Figure 2N) and δ power (Figure 2O) during isoflurane exposure, and increased δ power during recovery phase (Figure 2P). At 32°C T_a_, activation of LPO^BDNF^, neurons also showed similar results to the changes in that of 25°C T_a_ (Figures 2Q–2S and 2T–2W) but with no difference of RORR time (*n* = 6, *p* > 0.05; Figure 2T).

To determine whether the increased anesthetic depth resulted from reduced T_core_ or a direct effect of LPO^BDNF^ activation, we analyzed the temperature dependence of BSR and RORR time between CNO- and saline-treated groups (Figures 2X–2Y). BSR showed a linear correlation with mean T_core_ in both groups (Figure 2X), without significant differences in the regression slopes (*p* = 0.61) or intercepts (*p* = 0.08). Also, there is no change in the logarithmic inverse relationship between T_core_ and RORR time in the CNO and saline groups (Figure 2Y), suggesting that T_core_ is the primary determinant of BSR and RORR.

### Chemogenetic activation of DMH^Vglut2^ neurons mitigates anesthesia by increasing T_core_ and its intrinsic activity

**Figure 3:**
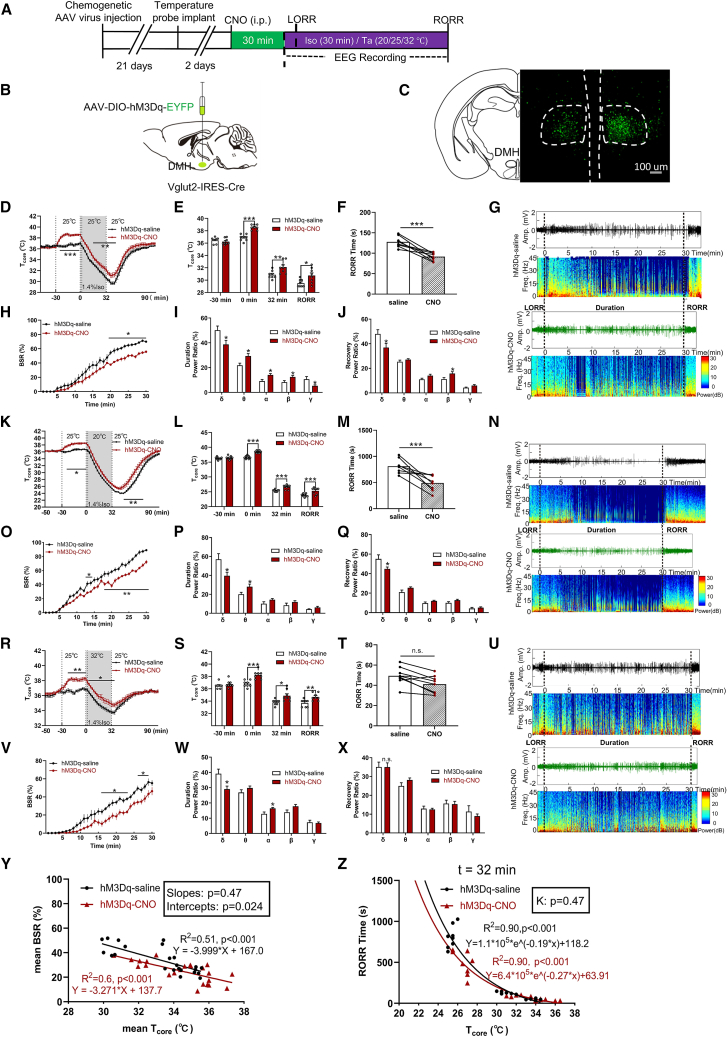
Chemogenetic activation of DMH^Vglut2^ mitigates isoflurane-induced anesthesia largely by increasing T_core_ (A) Protocol for chemogenetic experiment, CNO was intraperitoneally injected 30 min prior to isoflurane-induced anesthesia. T_core_ and EEG were monitored during the anesthesia procedure. (B) Schematic representation of *AAV-DIO-hM3D-EYFP* or *AAV-DIO-EYFP* injection into the DMH region of *Vglut2-ires-Cre* mice. (C) Representative images showing the expression of hM3Dq receptors (green) in the DMH of *Vglut2-ires-Cre* mice. Scale bars, 100 μm. (D) T_core_ changes at T_a_ of 25°C under isoflurane in hM3Dq-saline and hM3Dq-CNO mice. T_core_ was recorded every 2 min (measured by intraperitoneal implantation). CNO injected at −30 min, isoflurane started at *t* = 0 min, induced LORR of mice within 2 min, and maintained for 30 min, isoflurane ceased at *t* = 32 min. (E) The values of T_core_ at different time under isoflurane in hM3Dq-saline and hM3Dq-CNO mice. T_core_ at *t* = −30 min, the time of isoflurane starting (*t* = 0 min), isoflurane cessation (*t* = 32 min) and at the RORR moment (t_RORR_). (F) Chemogenetic activation of DMH^Vglut2^ neurons at T_a_ of 25°C shortened the RORR time. (G) Representative raw EEG traces (top) and EEG power spectra (bottom) at T_a_ of 25°C under isoflurane in hM3Dq-saline and hM3Dq-CNO mice. (H) BSR changes during isoflurane anesthesia procedure (30 min) at T_a_ of 25°C in hM3Dq-saline and hM3Dq-CNO mice. BSR is plotted at every minute. (I) DMH^Vglut2^ neural activation during isoflurane (0–30 min) displayed significant decrease in δ power band (F (4, 70) = 15.92; *p* = 0.031; *n* = 8). (J) DMH^Vglut2^ neural activation during recovery procedure (from isoflurane ceasing to RORR of mice) displayed significant decrease in δ power band (F (4, 70) = 19.26; *p* = 0.031; *n* = 8). (K) T_core_ changes at T_a_ of 20°C under isoflurane in hM3Dq-saline and hM3Dq-CNO mice. (L) The values of T_core_ at different time under isoflurane in hM3Dq-saline and hM3Dq-CNO mice. (M) Chemogenetic activation of DMH^Vglut2^ neurons shortened the RORR time. (N) Representative raw EEG traces (top) and EEG power spectra (bottom) at T_a_ of 20°C under isoflurane in hM3Dq-saline and hM3Dq-CNO mice. (O) BSR changes during isoflurane anesthesia procedure at T_a_ of 20°C in hM3Dq-saline and hM3Dq-CNO mice. (P) DMH^Vglut2^ neural activation during isoflurane anesthesia procedure (0–30 min) displayed significant decrease in δ power band (F (4, 70) = 19.89; *p* = 0.027; *n* = 8) and increase in θ power band (F (4, 70) = 4.18; *p* = 0.036; *n* = 8). (Q) DMH^Vglut2^ neural activation during recovery procedure (from isoflurane ceasing to RORR of mice) displayed significant decrease in δ power band (F (4, 70) = 15.59; *p* = 0.040; *n* = 8). (R) T_core_ changes at T_a_ of 32°C under isoflurane in hM3Dq-saline and hM3Dq-CNO mice. (S) The values of T_core_ at different time under isoflurane in hM3Dq-saline and hM3Dq-CNO mice. (T) Chemogenetic activation of DMH^Vglut2^ neurons has no influence on RORR time. (U) Representative raw EEG traces (top) and EEG power spectra (bottom) at T_a_ of 32°C under isoflurane in hM3Dq-saline and hM3Dq-CNO mice. (V) BSR changes during isoflurane anesthesia procedure at T_a_ of 32°C in hM3Dq-saline and hM3Dq-CNO mice. (W) DMH^Vglut2^ neural activation during isoflurane (0–30 min) displayed significant decrease in δ power band (F (4, 70) = 19.71; *p* = 0.014; *n* = 8) and increase in α power band (F (4, 70) = 2.55; *p* = 0.019; *n* = 8). (X) DMH^Vglut2^ neural activation during recovery procedure (from isoflurane ceasing to RORR of mice) displayed no significant differences. (Y) The linear regression model of mean BSR and mean T_core_ of hM3Dq-saline and hM3Dq-CNO mice during isoflurane procedure (30 min). hM3Dq-saline, Y = −3.999∗X + 167.0, *R*^2^ = 0.51, *p* < 0.001; hM3Dq-CNO, Y = −3.271∗X + 137.7, *R*^2^ = 0.6, *p* < 0.001. Comparation within groups: slopes, *p* = 0.47; intercepts, *p* = 0.023. (Z) Logarithmic regression model (one-phase decay) of RORR time as a function of T_core_ at the time of isoflurane ceasing (*t* = 32 min) in hM3Dq-saline and hM3Dq-CNO mice. hM3Dq-saline, Y = 1.1∗10^5^∗eˆ(−0.19∗x) +118.2, R^2^ = 0.90, *p* < 0.001, hM3Dq-CNO, Y = 6.4∗10^5^∗eˆ(−0.27∗x) +63.91, R^2^ = 0.90, *p* < 0.001. *k* value within groups comparation showed no differences (*p* = 0.47). All data are presented as mean ± SEM, ∗*p* < 0.05, ∗∗*p* < 0.01, and ∗∗∗*p* < 0.001, ns, not significant, (E), (L), and (S) were analyzed by multiple *t* test, (F), (M), and (T) were analyzed by paired *t* test, (H–J), (O–Q), and (V–X) were analyzed by two-way RM ANOVA followed by Bonferroni’s multiple comparisons test, (Y) was analyzed by linear regression analysis, and (Z) was analyzed by non-linear regression (one-phase decay) analysis.

Inspired by neural modulation in the LPO, we focused on DMH^Vglut2^ neurons, which are major downstream targets of LPO^BDNF^ neurons (∼53%, Figures S1C–S1F). Indeed, optogenetic activation of the axonal terminals of LPO^BDNF^ neurons in the DMH lowered T_core_ and increased BSR (Figures S1G–S1O). *Vglut2-ires-Cre* mice were used to label glutamate neurons (Figures S2A–S2D). We employed chemogenetic activation of DMH^Vglut2^ neurons by injecting AAV-Ef1α-DIO-hM3Dq-mCherry into the DMH (Figures 3A and 3B). Viral expression was clearly seen in the DMH (Figures 3C and S3A). CNO administration induced a ∼2°C increase in T_core_ (from 36.88°C ± 0.18°C to 38.56°C ± 0.15°C; Figures 3D and 3E), and this elevation persisted throughout anesthesia (0–32 min) and during RORR. At 25°C T_a_, T_core_ was significantly higher in the DMH^Vglut2^ neural activation mice at anesthesia cessation (32.13°C ± 0.35°C vs. 30.88°C ± 0.23°C, *p* = 0.0096) and the RORR moment (30.75°C ± 0.43°C vs. 29.50°C ± 0.21°C, *p* = 0.021; *n* = 8; Figures 3D and 3E), and RORR time was significantly shortened (hM3Dq-CNO 91.63 ± 9.55 s vs. hM3Dq-saline 127.50 ± 13.51 s; *n* = 8, *p* < 0.001; Figure 3F). EEG analysis revealed significant alterations in BSR and power spectra (Figure 3G). During isoflurane exposure, BSR (Figure 3H) and δ power (38.69% ± 3.32% vs. 50.10% ± 3.40%, *p* = 0.031; Figure 3I) showed significant reduction, and the decreased δ power also shown during recovery phase (Figure 3J). The same pattern changes were noted in the DMH^Vglut2^ neural activation mice at 20°C T_a_, such as decreased T_core_ reduction (Figures 3K and 3L), shortened RORR (Figure 3M), decreased BSR (Figures 3N–3O) and δ power (Figure 3P) during isoflurane exposure, and decreased δ power in recovery phase (Figure 3Q). At 32°C T_a_, activation of DMH^Vglut2^, neurons also showed similar results to the changes in that of 25°C T_a_ (Figures 3R and 3S, 3U–3W), also no difference shown in RORR time (*n* = 8, *p* > 0.05; Figure 3T) and EEG power bands during recovery (Figure 3X).

BSR in both the groups exhibited a linear relationship with mean T_core_ during anesthesia, showing as comparable slopes of the fitted lines (*p* = 0.47), while the intercepts of these lines differed significantly (*p* = 0.024; Figure 3Y). The temperature dependence of RORR time across the CNO and saline groups followed a logarithmic relationship with T_core_, and no significant differences were found between the fitted curves (*p* = 0.47; Figure 3Z). These data suggest that temperature is still the dominant modulatory factor for anesthesia depth after DMH^Vglut2^ activation, but its intrinsic neuronal activation also influences the isoflurane anesthesia.

### Chemogenetic inhibition of DMH^Vglut2^ neurons decreases T_core_ and deepens anesthesia

Conversely, we also performed chemogenetic inhibition to further define the role of DMH^Vglut2^ neurons in anesthesia regulation (Figure S3B). AAV-Ef1α-DIO-hM4Di-EYFP (hM4Di) or AAV-Ef1α-DIO-EYFP (EYFP) was injected into the DMH of *Vglut2-ires-Cre* mice (Figure S3C). After 3 weeks of viral expression, c-Fos staining confirmed robust neuronal inhibition in hM4Di-expressing mice following CNO administration (hM4Di-CNO: 17.33% ± 2.11% vs. hM4Di-saline: 4.00% ± 1.39%; *n* = 6; *p* < 0.001; Figure S3C). DMH^Vglut2^ neural inhibition induced hypothermia with a reduction of T_core_ by ∼2°C (37.50°C ± 0.20°C to 35.81 ± 0.16°C; Figures S3D and S3E). Under 1.4% isoflurane, this hypothermia was exacerbated, with significantly lower T_core_ at anesthesia cessation (29.56°C ± 0.15°C vs. 30.75°C ± 0.41°C; *p* = 0.017) and at RORR (28.81°C ± 0.27°C vs. 30.00°C ± 0.41°C; *p* = 0.029; *n* = 8; Figure S3E). Consistent with deeper anesthesia, hM4Di-CNO mice exhibited prolonged RORR time (142.25 ± 15.93 s vs. 120.25 ± 14.90 s; *n* = 8; *p* = 0.013; Figure S3F). EEG analysis (Figure S2G) demonstrated an elevated BSR (Figure S3H) and increased δ power during maintenance phase (Figure S3I). This increased δ power also occurred during recovery (Figure S3J).

### LPO^BNDF^ and DMH^Vglut2^ neuronal activity changes under isoflurane

**Figure 4:**
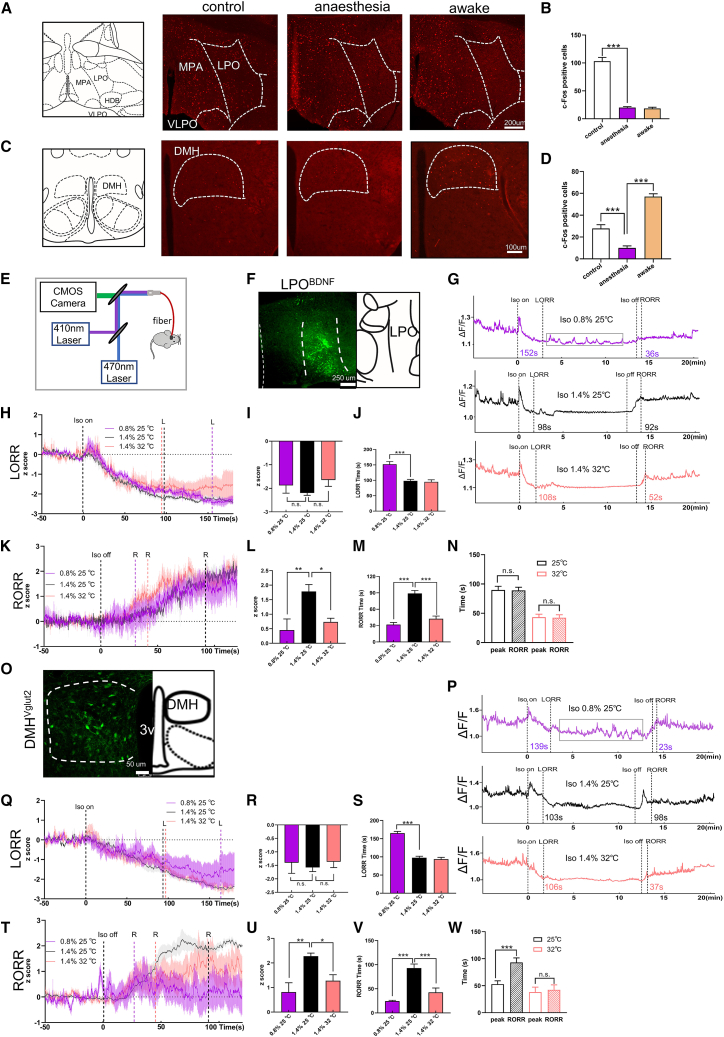
LPO^BDNF^ and DMH^Vglut2^ neuronal activity changes under isoflurane (A) Representative images showing the c-Fos expression (red) under neural state (left), anesthesia (middle) and awake (right) in LPO region. Scale bars, 200 μm. (B) Quantification of c-Fos expression in LPO in different anesthetic stages as indicated. (C and D) Representative images (C) and quantification (D) showing the c-Fos expression (red) under neural state (left), anesthesia (middle) and awake (right) in DMH region. Scale bars, 100 μm. (E) Schematic diagram of calcium signal recordings via fiber photometry. (F) Representative images showing the expression of *AAV2/9-hSyn-DIO-GCaMP6s-WPRE* virus in the LPO of *BDNF-ires-Cre* mice. Scale bars, 250 μm. (G) Representative raw Ca^2+^ signal trace of LPO^BDNF^ neurons in 0.8% isoflurane at 25°C (up), 1.4% isoflurane at 25°C (middle), and 32°C (bottom). (H and I) *Z* scored calcium ΔF/F traces of LPO^BDNF^ neurons (H) and quantification of the peak calcium *Z* scores (I) at LORR moment, isoflurane started at *t* = 0 s, the baseline is −50 to 0 s, and the dashed line represent the LORR moment of 0.8% isoflurane at 25°C (purple), 1.4% isoflurane at 25°C (black), and 32°C (pink). (J) LORR time of mice under different conditions. (K–L) *Z* scored calcium ΔF/F traces of LPO^BDNF^ neurons (K) and quantification of the peak calcium *Z* scores (L) at RORR moment after isoflurane ceasing (*t* = 0 s) under different conditions, and the dashed line represent the RORR moment of 0.8% isoflurane at 25°C (purple), 1.4% isoflurane at 25°C (black), and 32°C (pink). (M) RORR time of mice under different conditions. (N) RORR time and the peak time of Ca^2+^ signal after isoflurane ceasing at 25°C and 32°C T_a_. (O) Representative images showing the expression of *AAV2/9-hSyn-DIO-GCaMP6s-WPRE* virus in the DMH of *Vglut2-ires-Cre* mice. Scale bars, 100 μm. (P) Representative raw Ca^2+^ signal trace of DMH^Vglut2^ neurons in 0.8% isoflurane at 25°C (up), 1.4% isoflurane at 25°C (middle), and 32°C (bottom). (Q and R) *Z* scored calcium ΔF/F traces of DMH^Vglut2^ neurons (Q) and quantification of the peak calcium *Z* scores (R) at LORR moment, isoflurane started at *t* = 0 s, the baseline is −50 to 0 s, and the dashed line represent the LORR moment of 0.8% isoflurane at 25°C (purple), 1.4% isoflurane at 25°C (black), and 32°C (pink). (S) LORR time of mice under different conditions. (T and U) *Z* scored calcium ΔF/F traces of DMH^Vglut2^ neurons (T) and quantification of the peak calcium *Z* scores (U) at RORR moment after isoflurane ceasing (*t* = 0 s) under different conditions, and the dashed line represent the RORR moment of 0.8% isoflurane at 25°C (purple), 1.4% isoflurane at 25°C (black), and 32°C (pink). (V) RORR time of mice under different conditions. (W) RORR time and the peak time of Ca^2+^ signal after isoflurane ceasing at 25°C and 32°C T_a_. All data are presented as mean ± SEM, ∗*p* < 0.05, ∗∗*p* < 0.01, and ∗∗∗*p* < 0.001, n.s., not significant, (B), and (D) were analyzed by unpaired *t* test, and (I), (J), (L–M), (R), (S), and (U–W) were analyzed by one-way multiple comparisons test.

To determine the potential role of LPO and DMH neurons in anesthesia procedure, we examined c-Fos expression during isoflurane exposure, and anesthesia awake state separately. The LPO exhibited significantly lower c-Fos activation during both the awake and anesthesia stages (Figures 4A and 4B), while the DMH showed substantially greater c-Fos activation during the awake stage compared to the anesthesia stage (Figures 4C and 4D). These data are consistent with a possible direct and temperature-independent function of DMH in emergence.

Because c-Fos labeling provides limited temporal resolution of neural activity, we next directly recorded Ca^2+^ signal from LPO^BDNF^ and DMH^Vglut2^ neurons separately via fiber photometry (Figure 4E). Genetically encoded calcium indicators (GCaMPs) was selectively expressed in the LPO^BDNF^ (Figure 4F) and DMH^Vglut2^ neurons (Figure 4O). Isoflurane started at *t* = 0 and lasted for 10 min. The calcium signal of LPO^BDNF^ neurons revealed clear transient activation in the first 20 s of isoflurane exposure, and followed by a gradual decline (Figures 4G–4J). In the recovery process, the Ca^2+^ signals began to increase when isoflurane ceased (Figures 4K–4M), and peaked approximately at the moment of RORR (Figure 4N). These results suggest that LPO^BDNF^ neurons are not the primary direct target of isoflurane for inducing hypothermia and unconsciousness; rather, they modulate anesthetic depth secondarily through their influence on T_core_.

DMH^Vglut2^ neurons Ca^2+^ signal displayed similar suppression (Figures 4P–4S) to that of LPO^BDNF^ neurons under the anesthesia stage. In contrast to LPO, in the awake stage, DMH^Vglut2^ neurons displayed strong activity prior to the RORR time point (Figures 4T–4W), suggesting these neurons play an initiative role in driving emergence. This further implies that isoflurane-induced hypothermia and unconsciousness may be partially mediated by inhibiting this pro-arousal neural population.

### Stage-specific optogenetic activation of DMH^Vglut2^ neurons reveals its effect on anesthesia independent of temperature

**Figure 5:**
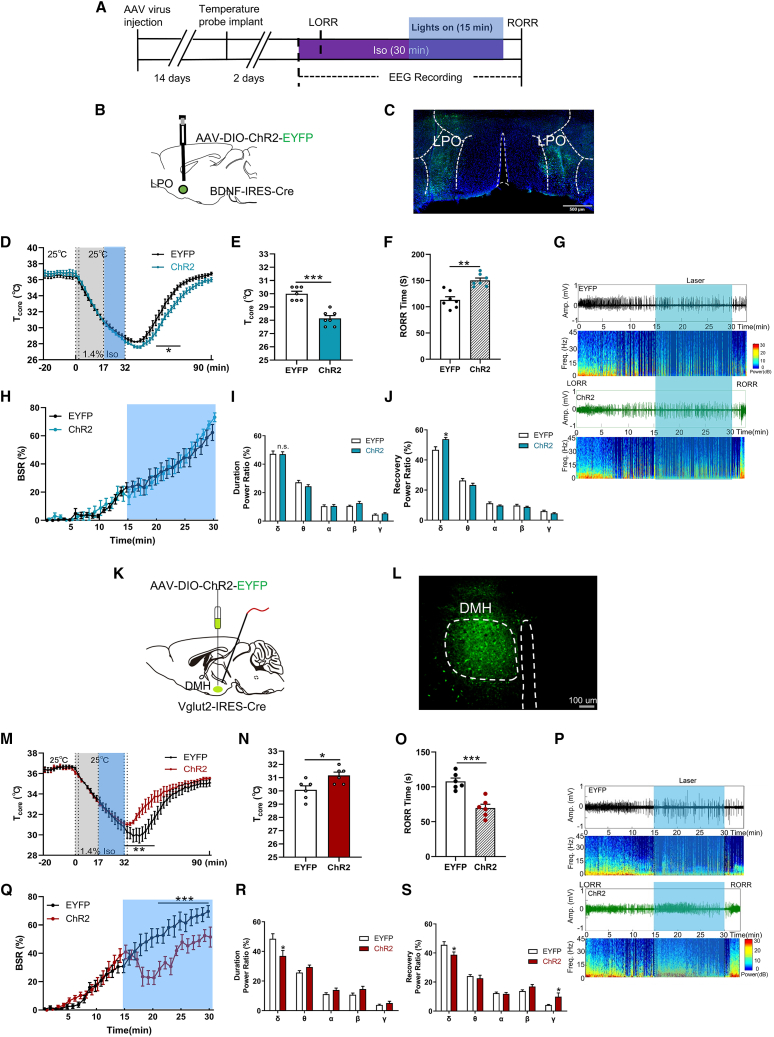
Stage-specific optogenetic activation of DMH^Vglut2^ neurons reveals its effect on anesthesia independent of temperature (A) Protocol for the optogenetic stimulation experiment. Blue shading indicates the optical stimulation phase, starting from 17 min after isoflurane induction and ending with isoflurane cessation at *t* = 32 min. (B) Schematic representation of *AAV-DIO-ChR2-EYFP* injection and optical fiber implantation into the LPO region of *BDNF-ires-Cre* mice. (C) Representative images showing the expression of ChR2-EYFP (green) in the LPO of *BDNF-ires-Cre* mice. Scale bars, 500 μm (right). (D) T_core_ changes of ChR2 and EYFP mice under isoflurane. T_core_ was recorded every 2 min. Isoflurane administration started at *t* = 0 min, maintained for 30 min and ceased at *t* = 32 min. Photo-stimulation started at *t* = 17 min and ceased at *t* = 32 min. (E) The values of T_core_ at the RORR moment under isoflurane in ChR2 and EYFP mice. (F) Optogenetic activation of LPO^BDNF^ neurons prolonged the RORR time. (G) Representative raw EEG traces (top) and EEG power spectra (bottom) at 25°C of T_a_ under isoflurane in ChR2 and EYFP mice. (H) BSR changes of ChR2 and EYFP mice during isoflurane anesthesia procedure (30 min). (I) LPO^BDNF^ neural activation during isoflurane (0–30 min) displayed no change. (J) LPO^BDNF^ neural activation during recovery procedure displayed significant increase in δ power band (F (4, 60) = 21.22; *p* = 0.011; *n* = 7). (K) Schematic representation of *AAV-DIO-ChR2-EYFP* or *AAV-DIO-EYFP* injection and optical fiber implantation into the DMH region of *Vglut2-ires-Cre* mice. (L) Representative images showing the expression of ChR2-EYFP (green) in the DMH of *Vglut2-ires-Cre* mice. Scale bars, 100 μm (right). (M) T_core_ changes of ChR2 and EYFP groups under isoflurane. (N) The values of T_core_ at the RORR moment under isoflurane in ChR2 and EYFP mice. (O) Optogenetic activation of DMH^Vglut2^ neurons shortened the RORR time of mice. (P) Representative raw EEG traces (top) and EEG power spectra (bottom) at 25°C of T_a_ under isoflurane in ChR2 and EYFP mice. (Q) BSR changes of ChR2 and EYFP mice during isoflurane anesthesia procedure (30 min). (R) DMH^Vglut2^ neural activation during isoflurane (0–30 min) displayed significant decrease in δ power band (F (4, 50) = 18.05; *p* = 0.035; *n* = 6). (S) DMH^Vglut2^ neural activation during recovery procedure (from isoflurane ceasing to RORR of mice) displayed significant decrease in δ power band (F (4, 50) = 9.36; *p* = 0.032; *n* = 6) and increase in γ power band (F (4, 50) = 6.59; *p* = 0.047; *n* = 6). All data are presented as mean ± SEM, ∗*p* < 0.05, ∗∗*p* < 0.01, and ∗∗∗*p* < 0.001, ns, not significant, (E), (F), (N), and (O) were analyzed by unpaired *t* test, and (H–J) and (Q–S) were analyzed by two-way RM ANOVA followed by Bonferroni’s multiple comparisons test.

We photoactivated LPO^BDNF^ and DMH^Vglut2^ neurons 15 min after LORR during isoflurane exposure for 15 min (Figure 5A). Optogenetic activation of LPO^BDNF^ neurons did not alter T_core_ during anesthesia (Figures 5B and 5C). EEG δ power and BSR also showed no change by photoactivation during anesthesia (Figures 5G–5I), suggesting undetected role in anesthetic depth. Another, similar changes showed when experiment was conducted at 20°C T_a_ (Figures S4A–S4F), but no changes in T_core_ and RORR at 32°C T_a_ (Figures S4G–S4L). Notably, photoactivation of LPO^BDNF^ neurons produced a post-stimulation effect, leading to reduced T_core_ during RORR at 25°C T_a_ (ChR2 28.25°C ± 0.21°C vs. EYFP 29.92°C ± 0.20°C, *p* < 0.001; Figures 5D and 5E), and prolonged RORR (ChR2 150.43 ± 11.86 s vs. EYFP 113.14 ± 16.05 s, *p* < 0.001, Figure 5F). The δ power was increased during recovery (ChR2 53.80% ± 1.16% vs. EYFP 46.71% ± 2.05%, *p* = 0.026; *n* = 7, Figure 5J). We propose that the post-stimulation effects likely arise from accumulated neuromodulators or a primed circuit state whose influence is unmasked after isoflurane withdrawal. Future studies using real-time neurotransmitter sensing or delayed antagonist application could directly test this priming model versus a slow neurohumoral release.

Conversely, DMH^Vglut2^ neural activation (Figures 5K and 5L) significantly increased T_core_ during RORR at 25°C T_a_ (−5.52°C ± 0.25°C vs. −6.97°C ± 0.53°C, *n* = 6; Figures 5M and 5N), which accelerated emergence from anesthesia (69.67 ± 5.41 s vs. 107.83 ± 4.83 s, *n* = 6, *p* < 0.001; Figures 5O–5S). Interestingly, EEG analysis revealed immediate BSR suppression following photo-stimulation (Figures 5P and 5Q) and reduced δ power without altering T_core_ during isoflurane exposure (36.95% ± 3.66% vs. 48.54% ± 3.38%, *n* = 6, *p* = 0.035; Figure 5R). Comparable effects on T_core_ and EEG parameters were observed at 20°C T_a_ (Figures S5A–S5F), while only BSR suppression showed in DMH^Vglut2^ neural activation mice at 32°C T_a_ (Figures S5G–S5L). Critically, under thermoneutral conditions (T_a_ = 36°C), where T_core_ was maintained, normothermia (T_core_ in between 36°C and 37°C) throughout the procedure, optogenetic activation of DMH^Vglut2^ neurons still elicited immediate BSR suppression without any change in T_core_ (Figures S5M–S5R). Collectively, these results demonstrate that DMH^Vglut2^ neurons exert a robust, direct wake-promoting effect during isoflurane anesthesia that is temperature-independent.

### The DMH^Vglut2^ to RPa pathway is essential for the temperature-independent effect on anesthesia regulation

**Figure 6:**
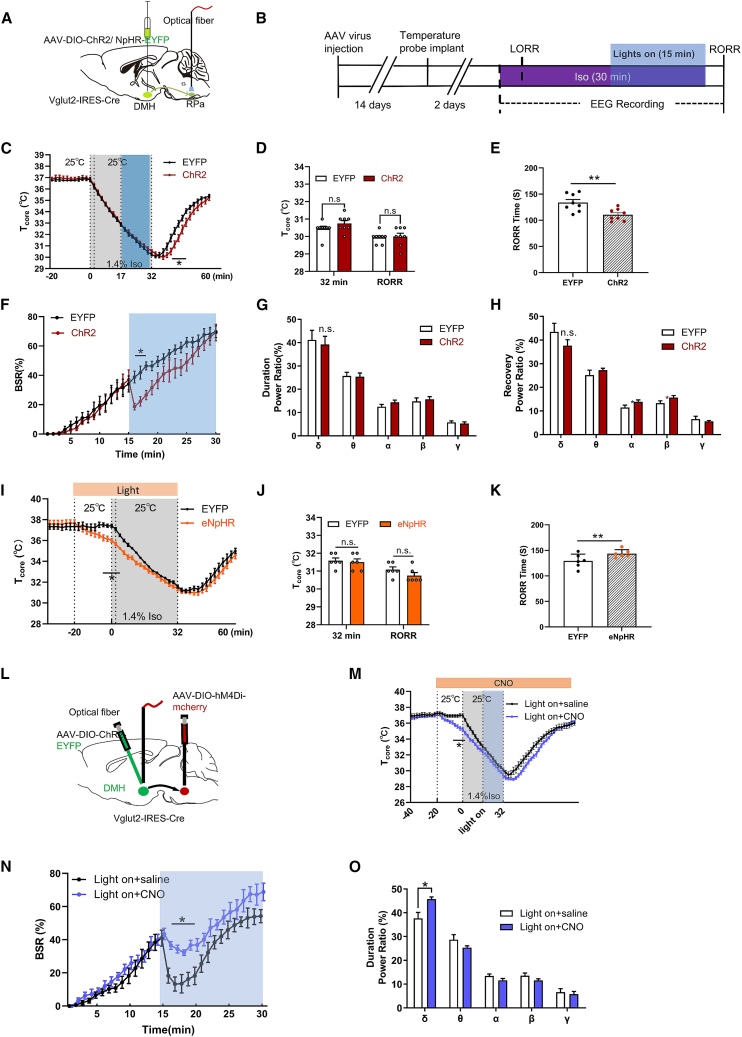
Optogenetic manipulation of the DMH^vglut2^ to RPa pathway reveals its temperature-independent effect on anesthesia (A) Schematic representation of *AAV-DIO-ChR2-EYFP (ChR2)* or *AAV-DIO-EYFP (EYFP)* injection into the DMH, and optical fiber implantation into the RPa of *Vglut2-ires-Cre* mice. (B) Protocol for the pathway optogenetic stimulation experiment. Blue shading indicates the optical stimulation phase, starting from 17 min after isoflurane induction and ending with isoflurane cessation at *t* = 32 min. (C) T_core_ changes of ChR2 and EYFP groups under isoflurane. (D) The values of T_core_ at different time under isoflurane in ChR2 and EYFP mice. T_core_ at the time of isoflurane cessation (*t* = 32 min), and at the RORR moment (t_RORR_). (E) Optogenetic activations of the DMH^Vglut2^ to RPa pathway shortened the RORR time. (F) BSR changes of ChR2 and EYFP mice during isoflurane anesthesia procedure. (G and H) Optogenetic activations of the DMH^Vglut2^ to RPa pathway displayed no significant changes in EEG power bands during isoflurane exposure (G), and recovery procedure (H). (I) T_core_ changes of eNpHR and EYFP mice under isoflurane. Photo-inhibition started at *t* = −20 min. (J) The values of T_core_ at different time under isoflurane in eNpHR and EYFP mice. (K) Optogenetic inhibition of the DMH^Vglut2^ to RPa pathway prolonged the RORR time. (L) Schematic representation of AAV-DIO-ChR2-EYFP virus and optical fiber implantation into the DMH region, and AAV-DIO-hM4Di-EYFP virus injection into RPa region of *Vglut2-ires-Cre* mice. (M) T_core_ changes of RPa-hM4Di mice (saline vs. CNO) with concurrent DMH^Vglut2^ activation under isoflurane. CNO injected at −20 min, isoflurane started at *t* = 0 min, induced LORR of mice within 2 min, and maintained for 30 min, isoflurane ceased at *t* = 32 min. (N) BSR changes of ChR2-saline and ChR2-CNO mice during isoflurane anesthesia procedure. (O) RPa^Vglut2^ neural inhibition increased δ power band (F (4, 60) = 17.8; *p* = 0.0024; *n* = 7) during isoflurane anesthesia procedure (0–30 min) in DMH^Vglut2^ neural activation mice. All data are presented as mean ± SEM, ∗*p* < 0.05, ∗∗*p* < 0.01, and ∗∗∗*p* < 0.001, ns, not significant, (D) and (J) were analyzed by multiple *t* test, (E) and (K) were analyzed by unpaired *t* test, and (F–H) and (N–O) were analyzed by two-way RM ANOVA followed by Bonferroni’s multiple comparisons test.

Building on the demonstrated temperature-independent effects of DMH^Vglut2^ neurons on anesthesia regulation, we sought to characterize the specific neural pathways mediating these effects. Given the established anatomical connection between DMH^Vglut2^ neurons and RPa under physiological conditions,^25^ we selectively manipulated this pathway by optogenetics (Figures 6A, 6B, and S6A–S6C). The pathway activation during isoflurane exposure neither affected T_core_ during anesthesia maintenance nor induced significant changes at RORR (Figures 6C and 6D). However, photo-stimulation significantly accelerated isoflurane recovery (ChR2 110.5 ± 11.39 s vs. EYFP 133.88 ± 16.75 s, *n* = 8, *p* < 0.001; Figure 6E) and transiently suppressed BSR (Figure 6F), while δ power remained unchanged (Figures 6G and 6H). The same pattern changes were noted in the pathway activation mice at 32°C T_a_ (Figures S6F–S6K). In the inhibition experiments, optogenetic suppression of DMH^Vglut2^ to RPa pathway only reduced T_core_ during the early stage of anesthesia (Figure 6I), with no differences observed at anesthesia cessation or RORR (Figure 6J). Further, inhibition significantly prolonged emergence time (eNpHR: 143.50 ± 7.74 s vs. EYFP 129.17 ± 13.62 s, *n* = 6, *p* = 0.0067; Figure 6K).

To validate the functional connection of the DMH^Vglut2^ to RPa projection, we performed additional experiments combining optogenetic activation of DMH^Vglut2^ and chemogenetic inhibition of the RPa (Figure 6L). In awake mice, this projection contributed to thermoregulation, as evidenced by a hyperthermia effect that was partially suppressed upon RPa inhibition (Figures S6D and S6E). Under isoflurane anesthesia, chemogenetic inhibition of RPa neurons (CNO injected at −20 min) showed an initial reduction of T_core_ (Figure 6M), and the mitigated anesthetic depth by DMH^Vglut2^ activation was also partially attenuated by RPa inhibition (Figures 6N and 6O). Collectively, these results demonstrate that the DMH^Vglut2^ to RPa pathway modulates anesthetic emergence independently of thermoregulatory mechanisms. And the arousal-promoting effect of DMH^Vglut2^ neurons is partly through Vglut2 neurons in RPa region.

## Discussion

Our study showed a linear correlation between T_a_ and T_core_ during anesthesia, and an exponential inverse correlation between T_core_ and anesthetic emergence, with a critical T_core_ threshold of ∼31.9°C. We speculate that a similar critical temperature threshold likely exists in humans, significantly influencing the timing of emergence from anesthesia. Elucidating such a threshold would hold important implications for clinical anesthesia management. Importantly, thermoregulatory neurons of LPO^BDNF^ and DMH^Vglut2^ were closely associated with anesthetic modulation. Specifically, while LPO^BDNF^ neurons mediated anesthetic effects inclusively through thermoregulation, DMH^Vglut2^ neurons exerted modulation via both temperature-dependent and temperature-independent mechanism. This work establishes a different paradigm for understanding how anesthesia is modulated by thermoregulatory neurons through defined neural circuit mechanisms, which are in some ways analogous to the sleep-wake circuits implicated in general anesthesia,^8^^,^^9^ thereby offering another insights into the mechanisms underlying anesthetic states.

The POA is a complex brain region composed of heterogeneous neuronal populations that regulate diverse functions and behaviors, including body temperature homeostasis,^26^^,^^27^ sleep-wake cycles,^28^^,^^29^ and anesthetics-induced states.^30^^,^^31^ BDNF protein is most expressed in the hippocampus and hypothalamus,^32^ and its expressing neurons are known to regulate feeding and energy expenditure.^33^^,^^34^ Recently, more studies have proved its thermoregulation function in the hypothalamus.^35^ BDNF neurons in MnPO were progressively activated during warm exposure, and optogenetic activation of those neurons resulted in hypothermia.^36^ Our data demonstrated that pre-anesthetic activation of LPO^BDNF^ neurons significantly decreased T_core_ and the hypothermic effect persisted throughout isoflurane exposure. However, the activation during isoflurane exposure did not induce body temperature change. This might be due to the impairment of behavioral regulation (e.g., shivering and vasoconstriction) induced by isoflurane.^2^ Previous studies reported that POA neurons (ventrolateral and median preoptic nucleus) modulated sleep-wake transitions, but do not influence general anesthesia-induced unconsciousness (mice with T_core_ maintained at 37°C–38°C).^37^ This is consistent with our results that LPO^BDNF^ neuronal optogenetic activation and chemogenetic activation at 32°C induced no change of anesthetic effect. However, the activation of LPO^BDNF^ neuron at room temperature conditions (20°C–25°C) increased anesthetic depth shown in our study, and this further supports the notion that LPO^BDNF^ neurons modulate anesthetic effect in a temperature-dependent way. While our experimental approach (e.g., acute optogenetic modulation) effectively captures immediate pharmacodynamic changes (like BSR suppression), it is not designed to dissect the intertwined roles of pharmacokinetics (e.g., isoflurane metabolism) and pharmacodynamics over longer periods such as recovery. A key future goal is to disentangle these effects, potentially through direct drug monitoring or closed-loop experimental paradigms. Optogenetic activation of the axonal terminals of LPO^BDNF^ neurons in the DMH lowered T_core_ and increased BSR during isoflurane, while whether LPO^BDNF^ neurons functioning through inhibiting DMH neurons needs further studies.

Activation of GABAergic or Vglut2-expressing neurons in the DMH contributed to hyperthermia.^15^^,^^36^^,^^38^ Our data showed that chemogenetic activation before isoflurane administration increased T_core_ during anesthesia procedure. We also found that optogenetic Vglut2 neurons during isoflurane suppressed BSR immediately and decreased δ power bands without altering T_core_, indicating its intrinsic consciousness regulation. It should be noted that although the stimulation parameters evoked robust responses, they may not capture the precise endogenous dynamics of these neuronal populations. Previous study reported that optogenetic activation of GABAergic neurons in DMH facilitate sevoflurane emergence.^24^ Although the DMH is a major downstream target of LPO, it also integrates inputs from other sleep-wake related nuclei, such as the ventromedial prefrontal cortex (vmPFC) and suprachiasmatic nucleus (SCN).^22^^,^^39^ This broader connectivity may explain its ability to influence anesthetic states independently of thermoregulation.

Previous studies demonstrated that DMH^Vglut2^ neurons project to the RPa, driving T_core_ elevation via brown adipose tissue (BAT) thermogenesis, shivering, and tail vasomotion.^16^^,^^38^^,^^40^ In line with these findings, we showed that optogenetic activation of the DMH^Vglut2^ to RPa pathway increased T_core_ during the awake state, but without altering T_core_ during isoflurane exposure. It remains to be determined whether isoflurane causes its effect through disruption of this neural pathway or through suppression of peripheral thermoregulatory functions. Intriguingly, under isoflurane anesthesia, this pathway modulates anesthetic depth, indicating a temperature-independent mechanism of isoflurane induced anesthesia. Although we demonstrated that the temperature-independent arousal-promoting effect of DMH^Vglut2^ neurons is partly mediated by Vglut2 neurons in the RPa, further investigation employing neuronal staining and cell-type-specific functional assays will be required to fully elucidate the diverse downstream effectors of this pathway. We also speculate that this temperature-independent effect may affect by elevated heart rate and blood pressure, as previous study showing that DMH-RPa pathway activation under anesthesia still raises both parameters.^41^ Given that the DMH^Vglut2^ neurons project to multiple downstream targets, including VTA and DR region,^22^ future studies are required to determine whether these complementary pathways also contribute to anesthetic state control, potentially forming a coordinated multi-synaptic network for arousal.

Our study may explain the high occurrence of hypothermia clinically, as the thermoregulatory system is profoundly affected by general anesthetics. Recent advances in targeted neuromodulation, including POA-mediated hypothermia induction in non-human primates^42^ and non-invasive T_core_ modulation via ultrasound^43^ highlight the translational potential of neural circuit-based temperature management. These approaches may inform the development of individualized anesthetic strategies that minimize the adverse effects of hypothermia while leveraging its potential advantages in some special medical conditions (e.g., cardiac surgery, organ transplantation, and neonatal encephalopathy^44^^,^^45^^,^^46^^,^^47^). Collectively, our findings provide critical insights into the neural mechanisms of isoflurane-induced hypothermia and general anesthesia.

### Limitations of the study

This study did not examine the peripheral vascular changes, which is regarded as another important factor that influences body temperature during anesthesia. Incorporating observations of the peripheral circulatory system, measuring BAT, monitoring T_core_ in brain or with higher sensitivity would provide more comprehensive understanding in further studies.^48^ The enhanced anesthetic effect observed under 20°C T_a_ may inevitably include neuroendocrine and metabolic alterations induced by cold stress.^49^^,^^50^ To avoid additional differences in gender,^51^ we prioritized male cohorts in experiments.

## Resource availability

### Lead contact

Further information and requests for resources should be directed to and will be fulfilled by the lead contact, shenwei@shanghaitech.edu.cn (Wei L. Shen).

### Materials availability

This study did not generate new unique reagents.

### Data and code availability

• All data reported in this paper will be shared by the lead contact upon request.
• This paper does not report original code.
• Any additional information required to reanalyze the data reported in this paper is available from the lead contact upon request.

## Acknowledgments

The authors thank Prof. Daqing Ma, Imperial College London, for critical comments during manuscript preparation. This work was supported by grants from 10.13039/100014718National Natural Science Foundation of China (no. 82430042 to T.Y.; nos. 32425028, 92357304, and 32330042 to W.L.S; nos. 82060653, and 82460707 to T.L.; no. 82160682 to S.Y.; and no. 32471076 to W.Z.Y.), 10.13039/501100013085Scientific and Technological Innovation 2030 under grant 2021ZD0204300. 10.13039/501100018537Noncommunicable Chronic Diseases-National Science and Technology Major Project (2024ZD0530300), the project (no. 2023-107 to W.Z.Y.) of Key Laboratory of Anesthesia and Organ Protection of Ministry of Education (In Cultivation), Zunyi Medical University. We thank the Shanghai Municipal Government and ShanghaiTech and Shanghai Frontiers Science Center for Bomacromolecules and Precision Medicine University for financial support.

## Author contributions

Study concept and design, W.L.S., T.Y., J.Z., S.C., and H.W.; acquisition of data, S.C., M.X., S.Y., H.C., and L.Z.; data and project management, W.Z.Y., T.L., and J.Z.; data cleaning and analysis, W.L.S., S.C., W.Z.Y., M.X., and K.Z.; drafting of the manuscript, S.C., W.L.S., W.Z.Y., J.Z., and T.Y. All authors participated in the interpretation and revision of critically important contents of the review. All authors approved the version of the manuscript submitted.

## Declaration of interests

The authors declare that they have no conflicts of interest.

## STAR★Methods

### Key resources table

**Table:** 

REAGENT or RESOURCE	SOURCE	IDENTIFIER
**Antibodies**		

Rabbit anti-*c*-Fos	Synaptic systems	Cat # 226003; RRID: AB_2231974
Rabbit anti-Glutamate	Sigma	Cat #G6642; RRID: AB_259946
Goat anti-rabbit IgG Alexa Fluor 594	Invitrogen	Cat # 111-585-144; RRID: AB_2307325

**Bacterial and virus strains**		

rAAV-Ef1α-DIO-hM3Dq-EYFP/mCherry	Brain Case	Cat BC-0145/0146
rAAV-Ef1α-DIO-hM4Di-EYFP	Brain Case	Cat BC-0154
rAAV-Ef1α-DIO-EYFP	Brain Case	Cat BC-0302
AAV-Ef1α-DIO-hChR2 (H134R)-EYFP	Brain Case	Cat BC-0107
AAV-Ef1α-DIO-eNpHR-EYFP	Brain Case	Cat BC-0125
AAV-hSyn-DIO-GCaMP6s	Brain Case	Cat BA-0238
rAAV-EF1α-DIO-WGA-Flpo	Brain Case	Cat BC-1400
rAAV-EF1α-FDIO-EGFP	Brain Case	Cat BC-0474

**Chemicals, peptides, and recombinant proteins**		

Isoflurane	RWD Life science	Cat R510-22-10
Clozapine-N-Oxide (CNO)	Sigma-Aldrich	Cat#C0832

**Software and algorithms**		

Thermochron iButton	Dallas Semiconductor	DS1922L
Wdsen	Wdsen Electronic Technology	http://wdsen.com/iButtonData.html
Spike2	Cambridge Electronic Design	https://ced.co.uk/upgrades/spike2demo
MATLAB	MathWorks	https://www.mathworks.com/products/matlab
GraphPad Prism 9.0	GraphPad Software	https://www.graphpad.com/

### Experimental model and study participant details

#### Animals

All experimental procedures were approved by the Animal Care and Use Committees of Zunyi Medical University, Guizhou, China ((2021) 2–581) and conducted in accordance with the ARRIVE guideline. Wild type adult C57BL/6 J mice (purchased from the Changsha Tianqin Biotechnology Co., Ltd, license number: SCXK2012-0005; Changsha, China), *Vglut2-ires-Cre* mice (JAX stock no. 016963), and *BDNF-ires-Cre* mice^52^ were used. All experiments were performed on male adult mice (8–16 weeks old). All animals were housed at an ambient temperature of 22 ± 2°C with a relative humidity of 60 ± 10% and a 12-h light/12-h dark cycle (light on at 8:00 p.m.). They freely access food and water.

### Method details

#### Stereotaxic brain surgeries and viral injection

Mice were anesthetized with pentobarbital (40 mg/kg, intraperitoneal [i.p.]) and then placed on a stereotaxic apparatus (RWD Life Science, Shenzhen, China). Lidocaine (1%) was subcutaneously injected before exposing the surface of the skull. The coordinates of viral injection sites include the LPO (AP, +0.5 mm; ML, ±0.82 mm; DV, −5.2 mm) and DMH (AP, −1.75 mm; ML, ±0.3 mm; DV, −5.1 mm). AAV virus was delivered (speed: 25 nL/min) through a glass micropipette (1-mm glass stock, tapering to a 10–20 microns tip) using a micro-syringe pump (Legato R130, KD Scientific, United States). The pipette was kept in the site for 10 min to allow the virus to diffuse before slowly withdrawn.

In chemogenetic experiments, virus was bilaterally and respectively injected 120 nL into the LPO of *BDNF-ires-Cre mice*, and DMH of *Vglut2-ires-Cre* mice. In optogenetics experiments, virus was unilaterally injected into LPO and DMH respectively, and the optical fibers (200 μm O.D., 0.37 numerical aperture, Newton Inc.) were positioned 0.2 mm above the injection site. The electroencephalographic electrodes were placed on the skull (AP: +1.0 mm, ML: ±1.5 mm; AP: −3.5 mm, ML: ±1 mm), and attached to the skull with a skull-penetrating screw and dental acrylic. In calcium signal recording experiments, virus was unilaterally and respectively injected into LPO and DMH, and optical fibers were positioned 0.2 mm above the injection site. During all the surgical procedures, a heating pad with a rectal temperature probe was used to keep the body temperature of mice around at 37°C. After surgery, a subcutaneous injection of carprofen (5 mg/kg) was administered for postoperative analgesia. Mice were then transferred to their housing cages for 3–4 weeks before next experiments.

#### Core body temperature recording

Abdominal temperature probe (Thermochron iButton DS1922L, Dallas Semiconductor, USA) was implanted into the mice under isoflurane anesthesia two days before experiments. After surgery, a subcutaneous injection of carprofen (5 mg/kg) was administered for postoperative analgesia. The data were recorded at every 2 min throughout experiments and analyzed offline (Wdsen Electronic Technology Co., Ltd, Shanghai).

#### EEG recording and analysis

Electroencephalography signals were captured during all the experiment with a neuronal recording system (Apollo, Bio-Signal Technologies, USA). These data were then digitized and analyzed using the Spike2 software package (Cambridge Electronic Design, Cambridge, United Kingdom). Delta (δ), theta (θ), alpha (α), beta (β), gamma (γ), and total spectral powers were calculated using the frequency bands of 1–4, 5–8, 9–12, 13–25, 26–60, and 1–60 Hz, respectively. Relative power within the frequency range of each band was divided by the total power in 1–60 Hz for data analysis. The burst suppression ratio (BSR) was the duration of the suppression wave divided by the total recording time, which computed with the MATLAB (MathWorks R2023a).

#### Ambient temperature controlling

A plastic water bag equipped with a thermostatic water was placed at the bottom of the anesthesia chamber to manipulate ambient temperature at 20°C, 25°C, 32°C, and 36°C during experiments.

#### Isoflurane exposure

Mice were placed in the anesthesia chamber (RWD Company, Shenzhen, China) and anesthetized with 1.4% isoflurane in oxygen enriched air at a rate of 1.5 L/min. We flipped the cage 90° every 15 s, the time of loss of righting reflex (LORR) and recovery of righting reflex (RORR) was determined when isoflurane exposure started and exposure stopped during ambient temperature at 20, 25, 32 or 36°C, respectively. All experiments were performed between 9:00 a.m. and 6:00 p.m.

In the chemogenetics groups, CNO (1 mg/mL, 1 mg/kg, i.p.) or saline (0.9%, equal volume, i.p.) were injected randomly 30 min before and then, EEG, LORR and RORR were recorded during and after isoflurane administration. In the optogenetics groups, the photo-stimulation (473 nm, 8 mW, 25 Hz, 10 ms duration, 1s off-cycle) (Thinker Tech, China) was conducted for 15 min, EEG, LORR and RORR were recorded during isoflurane administration.

#### Calcium fiber photometry recordings

AAV2/9-hSyn-DIO-GCaMP6s virus was injected and optical fibers (200 μm O.D., 0.37 numerical aperture, Inper Inc., China) was implemented in the LPO in the *BDNF-ires-Cre* mice and DMH of *Vglut2-ires-Cre* mice, respectively. The fluorescence signals were collected by a multichannel fiber photometry system (Inper Inc., China), equipped with a 488-nm and 410-nm lasers to collect signals and noise at a sampling rate of 50Hz, respectively, two weeks after injection, under 0.8% or 1.4% isoflurane exposure at 20, 25 or 32°C of ambient temperature, respectively, each for 10 min.

The values of fluorescence change (ΔF/F) were derived by calculating (F(t) − F)/F, where F is the baseline fluorescence signal averaged in a 50 s time window prior to the starting and ceasing of isoflurane, ΔF/F and *Z* score (Z(t) = (F(t) - μ)/σ), Z(t) = *Z* score at time t, F(t) = Raw fluorescence value at time t, μ = Mean baseline fluorescence, σ = Standard deviation of baseline fluorescence) represents changes in fluorescence.

#### Immunohistochemistry

Some cohort mice after above procedure or the hM3Dq and hM4Di mice injected with CNO for 2 h were killed with terminated pentobarbital anesthesia and perfused with the phosphate-buffered saline (PBS) followed by 4% paraformaldehyde (PFA). Their brains were removed and post-fixed in PFA overnight at 4°C and put in 30% sucrose in PBS at 4°C until they sank. The brains were coronally sectioned into 30-μm slices with a cryostat (Leica CM1950).

The sections were first incubated in blocking solution (PBS containing 2.5% normal goat serum, 1.5% bovine serum albumin, and 0.1% Triton X-100) for 2 h at room temperature. They were then incubated with the primary antibody (Rabbit anti-*c*-Fos, Synaptic systems, # 226003, 1:1000; Rabbit anti-Glutamate, Sigma, #G6642, 1:200) in a blocking solution overnight at 4°C and then with the related secondary antibody (Alexa Fluor 594 conjugated goat anti-rabbit IgG, Invitrogen, # 111-585-144, 1:1000) at room temperature for 2 h after washing with PBS. After washed with PBS, the sections were mounted on glass slides and cover-slipped with a mounting media (Gold antifade reagent with DAPI, Life Technologies, USA). All images were captured with the virtual microscopy system (Olympus BX63).

### Quantification and statistical analysis

All data were presented as mean ± SEM. Student’s t test were applied for statistical comparisons between the two groups, Multiple *t*-test and two-way ANOVA followed by Bonferroni’s multiple comparisons test were used in multiple groups comparison where appropriate. Linear regression analysis was used to analysis the relation of T_core_ and T_a_, T_core_ and LORR time, the BSR and T_core_. Non-linear regression (One phase decay) analysis was used to analysis the relation of T_core_ and RORR time. The statistical details are shown in results and figure legends. A significance threshold was set at *p* less than 0.05. All statistical analysis was carried out with the GraphPad Prism software (version 9.0, GraphPad Software Inc., San Diego, CA, USA).

## References

[1] Sessler D.I. (2016). Perioperative thermoregulation and heat balance. Lancet.

[2] Sessler D.I. (2008). Temperature monitoring and perioperative thermoregulation. Anesthesiology.

[3] Knaepel A. (2012). Inadvertent perioperative hypothermia: a literature review. J. Perioper. Pract..

[4] Giuliano K.K., Hendricks J. (2017). Inadvertent Perioperative Hypothermia: Current Nursing Knowledge. AORN J..

[5] Burger L., Fitzpatrick J. (2009). Prevention of inadvertent perioperative hypothermia. Br. J. Nurs..

[6] Yenari M.A., Han H.S. (2012). Neuroprotective mechanisms of hypothermia in brain ischaemia. Nat. Rev. Neurosci..

[7] Franks N.P., Zecharia A.Y. (2011). Sleep and general anesthesia. Can. J. Anaesth..

[8] Brown E.N., Purdon P.L., Van Dort C.J. (2011). General anesthesia and altered states of arousal: a systems neuroscience analysis. Annu. Rev. Neurosci..

[9] Bao W.W., Jiang S., Qu W.M., Li W.X., Miao C.H., Huang Z.L. (2023). Understanding the Neural Mechanisms of General Anesthesia from Interaction with Sleep-Wake State: A Decade of Discovery. Pharmacol. Rev..

[10] Kroeger D., Absi G., Gagliardi C., Bandaru S.S., Madara J.C., Ferrari L.L., Arrigoni E., Münzberg H., Scammell T.E., Saper C.B. (2018). Galanin neurons in the ventrolateral preoptic area promote sleep and heat loss in mice. Nat. Commun..

[11] Latifi B., Adamantidis A., Bassetti C., Schmidt M.H. (2018). Sleep-Wake Cycling and Energy Conservation: Role of Hypocretin and the Lateral Hypothalamus in Dynamic State-Dependent Resource Optimization. Front. Neurol..

[12] Harding E.C., Franks N.P., Wisden W. (2019). The Temperature Dependence of Sleep. Front. Neurosci..

[13] Tan C.L., Knight Z.A. (2018). Regulation of Body Temperature by the Nervous System. Neuron.

[14] Yang W.Z., Xie H., Du X., Zhou Q., Xiao Y., Zhao Z., Jia X., Xu J., Zhang W., Cai S. (2023). A parabrachial-hypothalamic parallel circuit governs cold defense in mice. Nat. Commun..

[15] Zhao Z.D., Yang W.Z., Gao C., Fu X., Zhang W., Zhou Q., Chen W., Ni X., Lin J.K., Yang J. (2017). A hypothalamic circuit that controls body temperature. Proc. Natl. Acad. Sci. USA.

[16] Yoshida K., Li X., Cano G., Lazarus M., Saper C.B. (2009). Parallel preoptic pathways for thermoregulation. J. Neurosci..

[17] Wanner S.P., Almeida M.C., Shimansky Y.P., Oliveira D.L., Eales J.R., Coimbra C.C., Romanovsky A.A. (2017). Cold-Induced Thermogenesis and Inflammation-Associated Cold-Seeking Behavior Are Represented by Different Dorsomedial Hypothalamic Sites: A Three-Dimensional Functional Topography Study in Conscious Rats. J. Neurosci..

[18] Morrison S.F. (2016). Central neural control of thermoregulation and brown adipose tissue. Auton. Neurosci..

[19] Piñol R.A., Zahler S.H., Li C., Saha A., Tan B.K., Škop V., Gavrilova O., Xiao C., Krashes M.J., Reitman M.L. (2018). Brs3 neurons in the mouse dorsomedial hypothalamus regulate body temperature, energy expenditure, and heart rate, but not food intake. Nat. Neurosci..

[20] Li L., Zhang M.Q., Sun X., Liu W.Y., Huang Z.L., Wang Y.Q. (2022). Role of Dorsomedial Hypothalamus GABAergic Neurons in Sleep-Wake States in Response to Changes in Ambient Temperature in Mice. Int. J. Mol. Sci..

[21] Cao W.H., Morrison S.F. (2006). Glutamate receptors in the raphe pallidus mediate brown adipose tissue thermogenesis evoked by activation of dorsomedial hypothalamic neurons. Neuropharmacology.

[22] Deurveilher S., Semba K. (2005). Indirect projections from the suprachiasmatic nucleus to major arousal-promoting cell groups in rat: implications for the circadian control of behavioural state. Neuroscience.

[23] Scammell T.E., Arrigoni E., Lipton J.O. (2017). Neural Circuitry of Wakefulness and Sleep. Neuron.

[24] Wang Y., Song Y., Tong L., Wang L., Cao J., Qin G., Liu X., Mi W., Wang E., Guo Y. (2023). GABAergic neurons in the dorsomedial hypothalamus regulate states of consciousness in sevoflurane anesthesia. iScience.

[25] Wu J., Liu D., Li J., Sun J., Huang Y., Zhang S., Gao S., Mei W. (2022). Central Neural Circuits Orchestrating Thermogenesis, Sleep-Wakefulness States and General Anesthesia States. Curr. Neuropharmacol..

[26] Morrison S.F., Nakamura K. (2019). Central Mechanisms for Thermoregulation. Annu. Rev. Physiol..

[27] Nakamura K. (2011). Central circuitries for body temperature regulation and fever. Am. J. Physiol. Regul. Integr. Comp. Physiol..

[28] Gallopin T., Fort P., Eggermann E., Cauli B., Luppi P.H., Rossier J., Audinat E., Mühlethaler M., Serafin M. (2000). Identification of sleep-promoting neurons in vitro. Nature.

[29] McGinty D., Szymusiak R. (2001). Brain structures and mechanisms involved in the generation of NREM sleep: focus on the preoptic hypothalamus. Sleep Med. Rev..

[30] Yatziv S.L., Yudco O., Dickmann S., Devor M. (2020). Patterns of neural activity in the mouse brain: Wakefulness vs. General anesthesia. Neurosci. Lett..

[31] Han B., McCarren H.S., O'Neill D., Kelz M.B. (2014). Distinctive recruitment of endogenous sleep-promoting neurons by volatile anesthetics and a nonimmobilizer. Anesthesiology.

[32] Nawa H., Carnahan J., Gall C. (1995). BDNF protein measured by a novel enzyme immunoassay in normal brain and after seizure: partial disagreement with mRNA levels. Eur. J. Neurosci..

[33] Liao G.Y., An J.J., Gharami K., Waterhouse E.G., Vanevski F., Jones K.R., Xu B. (2012). Dendritically targeted Bdnf mRNA is essential for energy balance and response to leptin. Nat. Med..

[34] Machado N.L.S., Saper C.B. (2022). Genetic identification of preoptic neurons that regulate body temperature in mice. Temperature (Austin).

[35] Chen B., Gao C., Liu C., Guo T., Hu J., Xue J., Tang K., Chen Y., Yu T., Shen Q. (2025). Heat acclimation in mice requires preoptic BDNF neurons and postsynaptic potentiation. Cell Res..

[36] Tan C.L., Cooke E.K., Leib D.E., Lin Y.C., Daly G.E., Zimmerman C.A., Knight Z.A. (2016). Warm-Sensitive Neurons that Control Body Temperature. Cell.

[37] Vanini G., Bassana M., Mast M., Mondino A., Cerda I., Phyle M., Chen V., Colmenero A.V., Hambrecht-Wiedbusch V.S., Mashour G.A. (2020). Activation of Preoptic GABAergic or Glutamatergic Neurons Modulates Sleep-Wake Architecture, but Not Anesthetic State Transitions. Curr. Biol..

[38] Machado N.L.S., Abbott S.B.G., Resch J.M., Zhu L., Arrigoni E., Lowell B.B., Fuller P.M., Fontes M.A.P., Saper C.B. (2018). A Glutamatergic Hypothalamomedullary Circuit Mediates Thermogenesis, but Not Heat Conservation, during Stress-Induced Hyperthermia. Curr. Biol..

[39] Zhong H., Xu H., Li X., Xie R.G., Shi Y., Wang Y., Tong L., Zhu Q., Han J., Tao H. (2023). A role of prefrontal cortico-hypothalamic projections in wake promotion. Cereb. Cortex.

[40] Sarkar S., Zaretskaia M.V., Zaretsky D.V., Moreno M., DiMicco J.A. (2007). Stress- and lipopolysaccharide-induced c-fos expression and nNOS in hypothalamic neurons projecting to medullary raphe in rats: a triple immunofluorescent labeling study. Eur. J. Neurosci..

[41] Kataoka N., Hioki H., Kaneko T., Nakamura K. (2014). Psychological stress activates a dorsomedial hypothalamus-medullary raphe circuit driving brown adipose tissue thermogenesis and hyperthermia. Cell Metab..

[42] Zhang Z., Shan L., Wang Y., Li W., Jiang M., Liang F., Feng S., Lu Z., Wang H., Dai J. (2023). Primate preoptic neurons drive hypothermia and cold defense. Innovation.

[43] Yang Y., Yuan J., Field R.L., Ye D., Hu Z., Xu K., Xu L., Gong Y., Yue Y., Kravitz A.Y. (2023). Induction of a torpor-like hypothermic and hypometabolic state in rodents by ultrasound. Nat. Metab..

[44] Melrose D.G., Dreyer B., Bentall H.H., Baker J.B. (1955). Elective cardiac arrest. Lancet.

[45] Tveita T., Sieck G.C. (2022). Physiological Impact of Hypothermia: The Good, the Bad, and the Ugly. Physiology.

[46] Baumann E., Preston E., Slinn J., Stanimirovic D. (2009). Post-ischemic hypothermia attenuates loss of the vascular basement membrane proteins, agrin and SPARC, and the blood-brain barrier disruption after global cerebral ischemia. Brain Res..

[47] Kirkegaard H., Søreide E., de Haas I., Pettilä V., Taccone F.S., Arus U., Storm C., Hassager C., Nielsen J.F., Sørensen C.A. (2017). Targeted Temperature Management for 48 vs 24 Hours and Neurologic Outcome After Out-of-Hospital Cardiac Arrest: A Randomized Clinical Trial. JAMA.

[48] Zhou Q., Fu X., Xu J., Dong S., Liu C., Cheng D., Gao C., Huang M., Liu Z., Ni X. (2023). Hypothalamic warm-sensitive neurons require TRPC4 channel for detecting internal warmth and regulating body temperature in mice. Neuron.

[49] Xu X., Tikuisis P. (2014). Thermoregulatory modeling for cold stress. Compr. Physiol..

[50] Willner P. (2005). Chronic mild stress (CMS) revisited: consistency and behavioural-neurobiological concordance in the effects of CMS. Neuropsychobiology.

[51] Zhang Z., Reis F.M.C.V., He Y., Park J.W., DiVittorio J.R., Sivakumar N., van Veen J.E., Maesta-Pereira S., Shum M., Nichols I. (2020). Estrogen-sensitive medial preoptic area neurons coordinate torpor in mice. Nat. Commun..

[52] Luo F., Mu Y., Gao C., Xiao Y., Zhou Q., Yang Y., Ni X., Shen W.L., Yang J. (2019). Whole-brain patterns of the presynaptic inputs and axonal projections of BDNF neurons in the paraventricular nucleus. J. Genet. Genomics..

