# Supplementary material for: Hypothalamic thermoregulatory neurons divergently modulate isoflurane anesthesia via temperature dependent and independent mechanisms
Source: iScience. 2026 Mar 30;29(5):115542. doi: 10.1016/j.isci.2026.115542 (PMC13091562; doi:10.1016/j.isci.2026.115542)
Supplement: Document S1. Figures S1–S6 [file mmc1.pdf]

iScience, Volume *29*

Supplemental information

Hypothalamic thermoregulatory neurons divergently modulate isoflurane anesthesia via temperature dependent and independent mechanisms

Shuang Cai, Wen Z. Yang, Mao Xu, Huimin Chen, Liang Zhou, Tianyuan Luo, Shouyang Yu, Kan Zhang, Haiying Wang, Tian Yu, Jijian Zheng, and Wei L. Shen

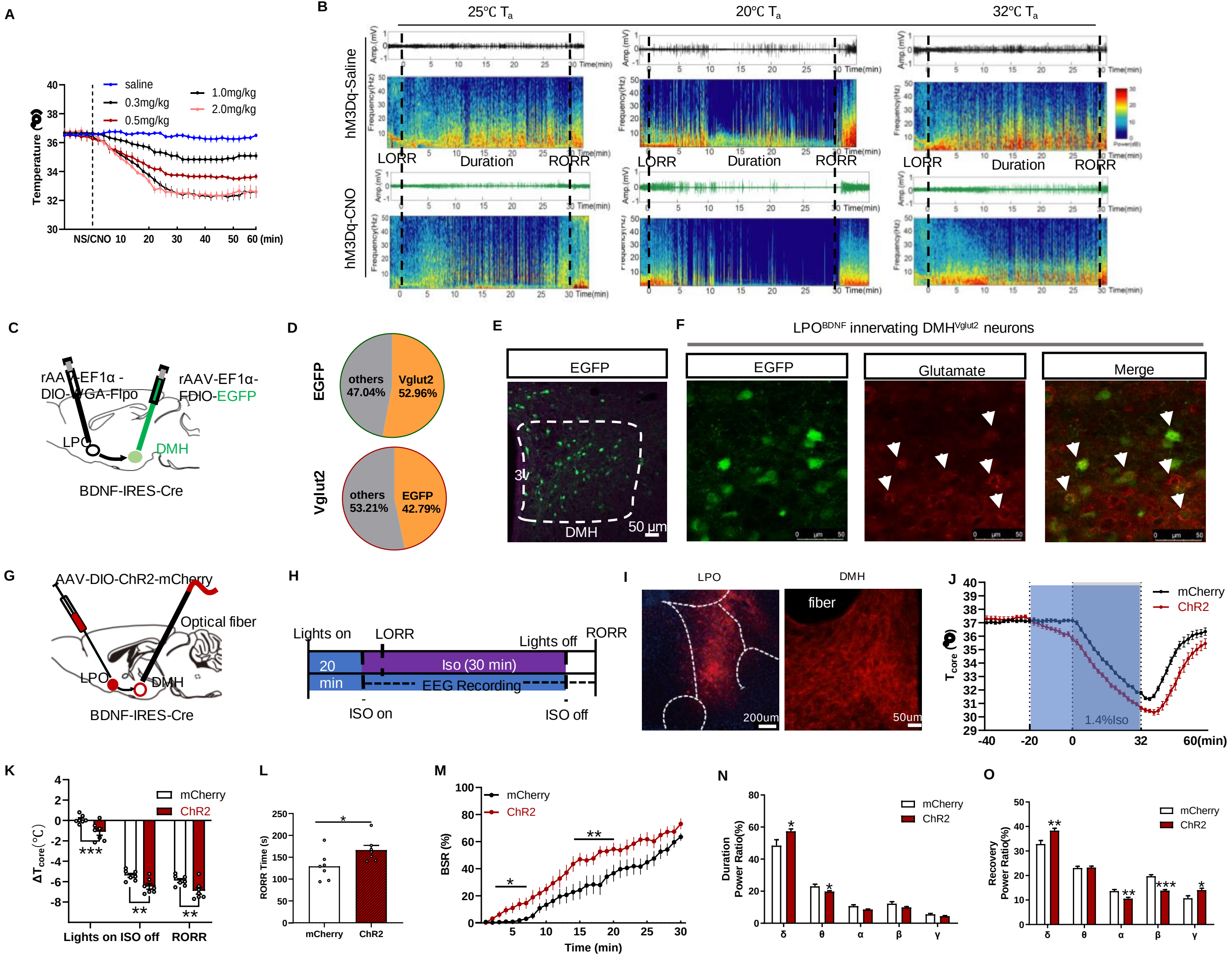

Figure S1. Supplemental EEG signals in LPO^{BDNF} neurons activation and functional validation of LPO^{BDNF}-DMH pathway

(A) Decreasing of T_{core} induced by different dosage of CNO injection in 60 min. (B) Representative raw EEG traces (top) and EEG power spectra (bottom) under isoflurane in hM3Dq-saline and hM3Dq-CNO mice at 25℃ (left), 20℃ (middle), and 32℃ (right) T_a. (C) Schematic representation of *rAAV-EF1α-DIO-WGA-Flpo* virus in LPO region and *rAAV-EF1α-FDIO-EGFP* virus in DMH region of *Vglut2-ires-Cre* mice. (D) Pie chart showing glutamatergic neurons percentage in LPO^{BDNF} to DMH neurons (top), and LPO^{BDNF} projecting neurons percentage in DMH^{Vglut2} neurons (bottom). (E) Representative images showing the virus expression in the DMH. Scale bars, 50 μm. (F) Representative images showing the LPO^{BDNF} innervating DMH neurons (green) and glutamate staining (red) in the DMH. Scale bars, 50 μm. (G) Schematic representation of *AAV-DIO-ChR2-mCherry* injection into the LPO and optical fiber implantation into DMH of *BDNF-Cre* mice. (H) Schematic of the optogenetic activation experiments protocol. (I) Representative images showing the expression of ChR2 receptors (red) in LPO (Scare bars, 200 μm) and DMH (Scale bars, 100 μm). (J) T_{core} changes under isoflurane in ChR2 and mCherry mice. (K) The mean changes of T_{core} under isoflurane in ChR2 and mCherry mice (mean of T_{core} from t_{-40}-t_{-20} as baseline, T_{core} at t_0 minus baseline means photo-stimulation induced change), the time of isoflurane ceasing (T_{core} at t_{32} minus baseline), and RORR moment (T_{core} at t_{RORR} minus baseline). (L) Optogenetic activation of LPO^{BDNF}-DMH pathway prolonged the RORR time (166.57 ± 10.62 s *vs*. 129.29 ±12.16 s, p = 0.039, n = 7). (M) BSR during isoflurane anesthesia procedure in ChR2 and mCherry mice. (N) LPO^{BDNF}-DMH pathway activation during isoflurane displaying significant increase in δ power band (F (4,60) = 19.37; p = 0.04; n = 7) and decrease in θ power band F (4,60) = 2.64; p = 0.034; n = 7). (O) LPO^{BDNF}-DMH pathway activation during recovery procedure displaying significant increase in δ (F (4,60) = 22.56; p = 0.0095; n = 7) and γ (F (4,60) = 8.35; p = 0.026; n = 7) power band and decrease in α (F (4,60) = 7.14; p = 0.0052; n = 7) and β (F (4,60) = 26.63; p < 0.001; n = 7) power band. All data are presented as mean ± SEM, *p<0.05, **p<0.01, and ***p<0.001.

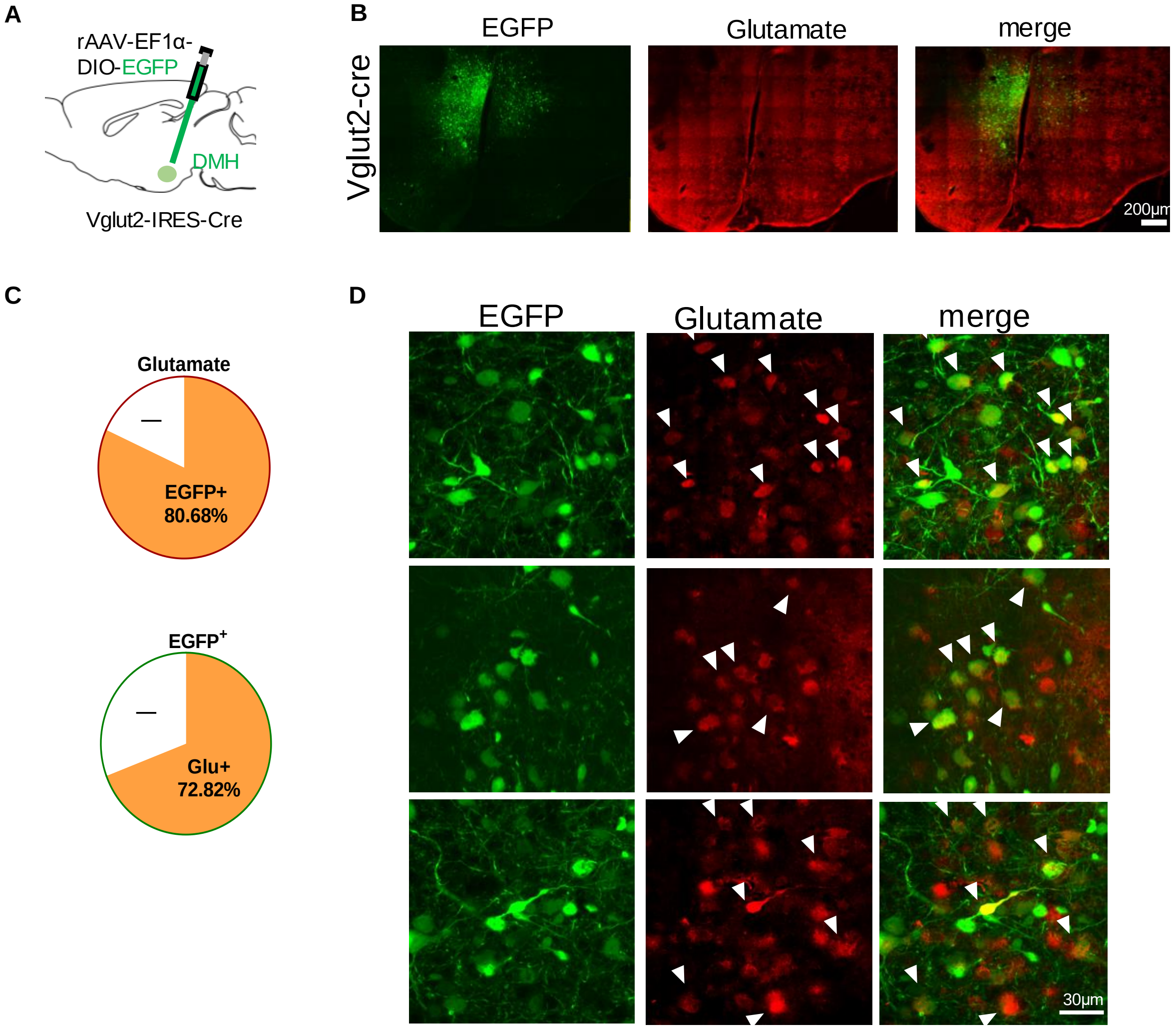

Figure S2 Vglut2 neurons in the DMH

(A) Schematic representation of *rAAV-EF1α-DIO-EGFP* virus in DMH region of *Vglut2-ires-Cre* mice.

(B) Representative images showing the expression of EGFP (green) and glutamate stanning (red) in the DMH of *Vglut2-ires-Cre* mice. Scare bar, 200 μm.

(C) Proportion of glutamate stanning of vglut2 neurons in DMH.

(D) Representative images showing the expression of EGFP (green) and glutamate stanning (red) in the DMH of *Vglut2-ires-Cre* mice. Scare bar, 30 μm.

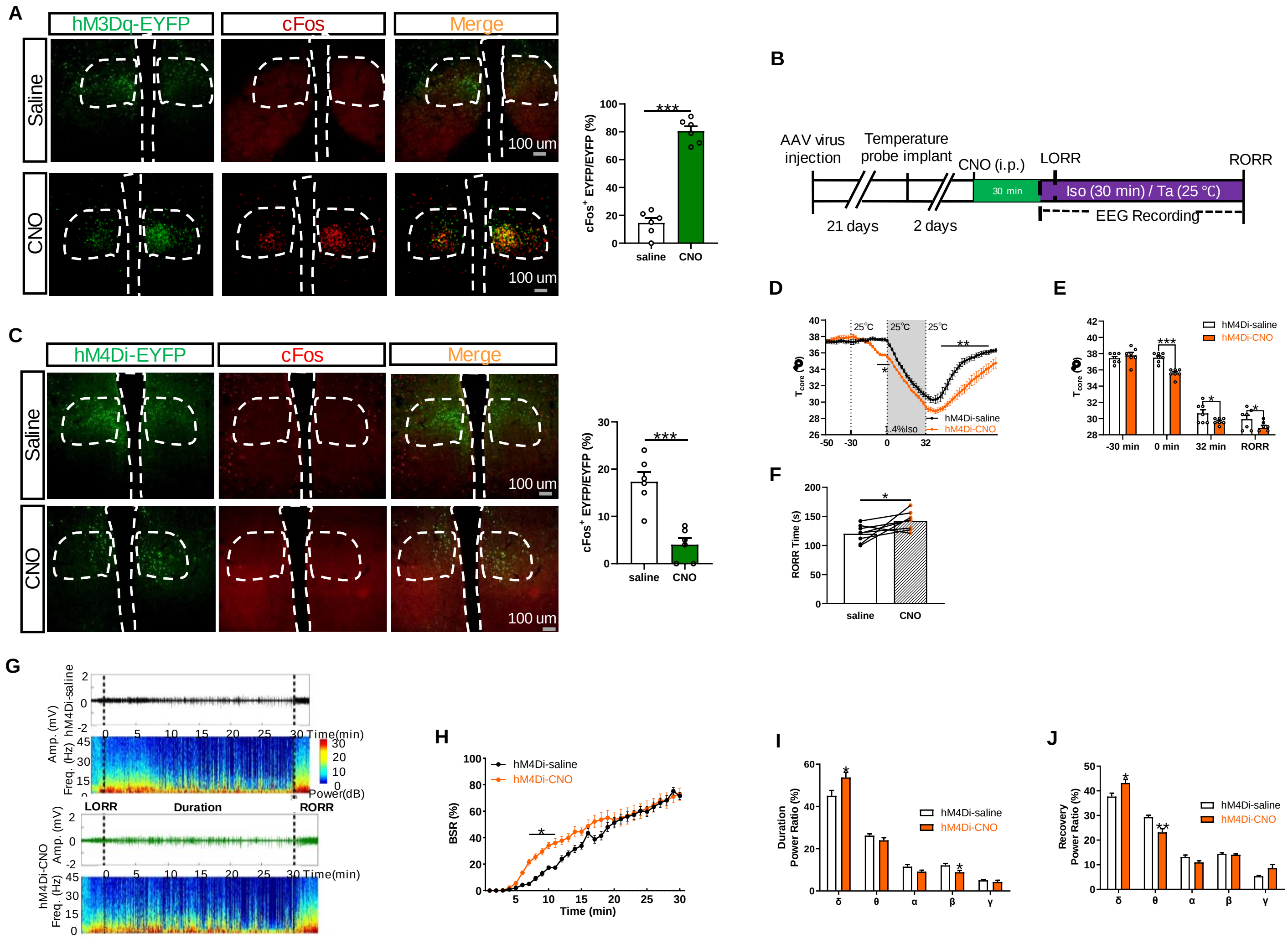

Figure S3. Expression of DMH chemogenetic viruses in the DMH and related behavior tests

(A) Representative images showing the expression of *hM3Dq-EYFP* (green) and c-Fos (red) in the DMH of *Vglut2-ires-Cre* mice after injection of saline (top) and CNO (bottom). Scale bars, 100 μm (left). Quantification of mean numbers of c-Fos expression in hM3Dq-saline and hM3Dq-CNO mice (right, 80.5 ± 3.56 % vs. 14.5 ± 3.58 %, $p < 0.001$, n = 6). (B) Protocol for chemogenetic experiment, CNO was intraperitoneally injected 30 min prior to isoflurane-induced anesthesia. (C) Representative images showing the expression of hM4Di receptors (green) and c-Fos (red) in the DMH of *Vglut2-ires-Cre* mice after injection of saline (top) and CNO (bottom). Scale bars, 100 μm (left). Quantification of mean numbers of c-Fos expression in hM4Di-saline and hM4Di-CNO mice (right, 4.0 ± 1.39 % vs. 17.33 ± 2.11 %, $p < 0.001$, n = 6). (D) T_{core} changes under isoflurane in hM4Di-saline and hM4Di-CNO mice. (E) The values of T_{core} at different time under isoflurane in hM4Di-saline and hM4Di-CNO mice. (F) Chemogenetic inhibition of DMH^{Vglut2} neurons prolonged the RORR time (142.25 ± 15.93 s *vs*. 120.25 ±14.89 s, p = 0.040, n = 7). (G) Representative raw EEG traces (top) and EEG power spectra (bottom) under isoflurane in hM4Di-saline and hM4Di-CNO mice. (H) BSR during isoflurane anesthesia procedure (30 min) in hM4Di-saline and hM4Di-CNO mice. (I) DMH^{Vglut2} neural inhibition during isoflurane (0-30 min) displayed significant increase in δ power band ($F_{(4,60)}$ = 20.88; p = 0.026; n = 7) and decrease in β power band ($F_{(4,60)}$ = 2.88; p = 0.021; n = 7). (J) DMH^{Vglut2} neural inhibition during recovery procedure (from isoflurane ceasing to RORR of mice) displayed significant increase in δ and power band ($F_{(4,60)}$ = 14.92; p = 0.015; n = 7) and decrease in θ power band ($F_{(4,60)}$ = 19.04; p = 0.0023; n = 7). All data are presented as mean ± SEM, *p<0.05, **p<0.01, and ***p<0.001.

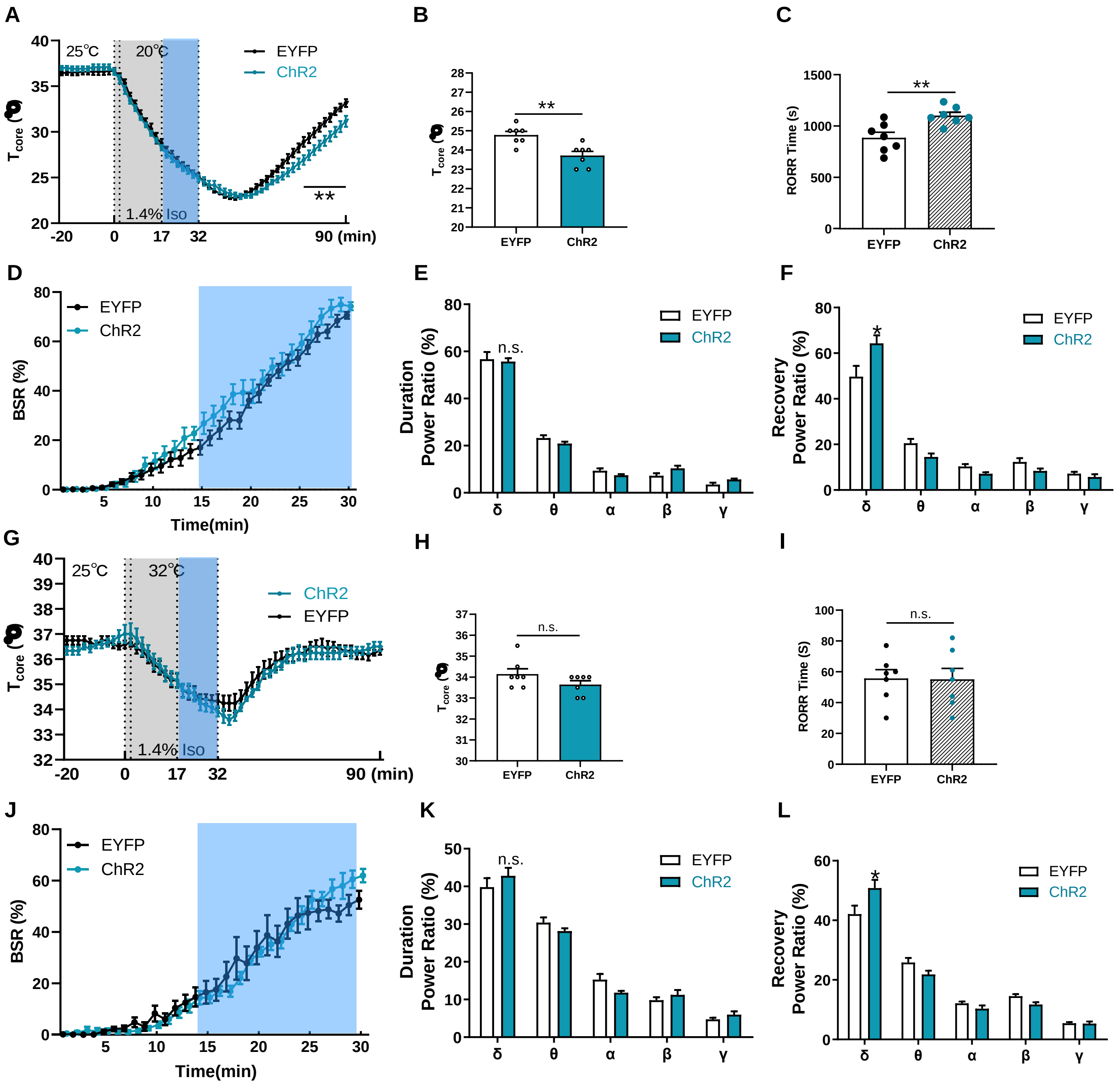

Figure S4. Stage-specific optogenetic activation of LPO^BDNF neurons under different conditions.

(A-B) T_{core} changes during isoflurane (A) and the values at RORR moment (B) in LPO^{BDNF} neurons activation mice at 20℃ of T_a. (23.71 ± 0.21 ℃ *vs*. 24.79 ±0.18 ℃, p = 0.0026, n = 7).

(C) LPO^{BDNF} neural activation during isoflurane exposure prolonged the RORR time at 20℃ of T_a (1101.28 ± 32.64 s *vs*. 886.14 ±53.23 s, p = 0.0048, n = 7).

(D) BSR during isoflurane anesthesia procedure (30 min) in LPO^{BDNF} neurons activation mice at 20℃ of T_a.

(E-F) LPO^{BDNF} neural activation showed no difference during isoflurane exposure (E), but increased δ power band ($F_{(4,60)} = 22.26$; $p = 0.025$; n = 7) during recovery (F) at 20℃ of T_a.

(G-H) T_{core} changes during isoflurane (G) and the values at RORR moment (H) in LPO^{BDNF} neurons activation mice at 32℃ of T_a.

(I) LPO^{BDNF} neural activation during isoflurane exposure showed no difference in RORR time at 32℃ of T_a.

(J) BSR during isoflurane anesthesia procedure (30 min) in LPO^{BDNF} neurons activation mice at 32℃ of T_a.

(K-L) LPO^{BDNF} neural activation showed no difference during isoflurane exposure (K), but increased δ power band ($F_{(4,60)} = 16.85$; $p = 0.04$; n = 7) during recovery (L) at 32℃ of T_a.

All data are presented as mean ± SEM, *p<0.05, **p<0.01, and ***p<0.001, ns, not significant.

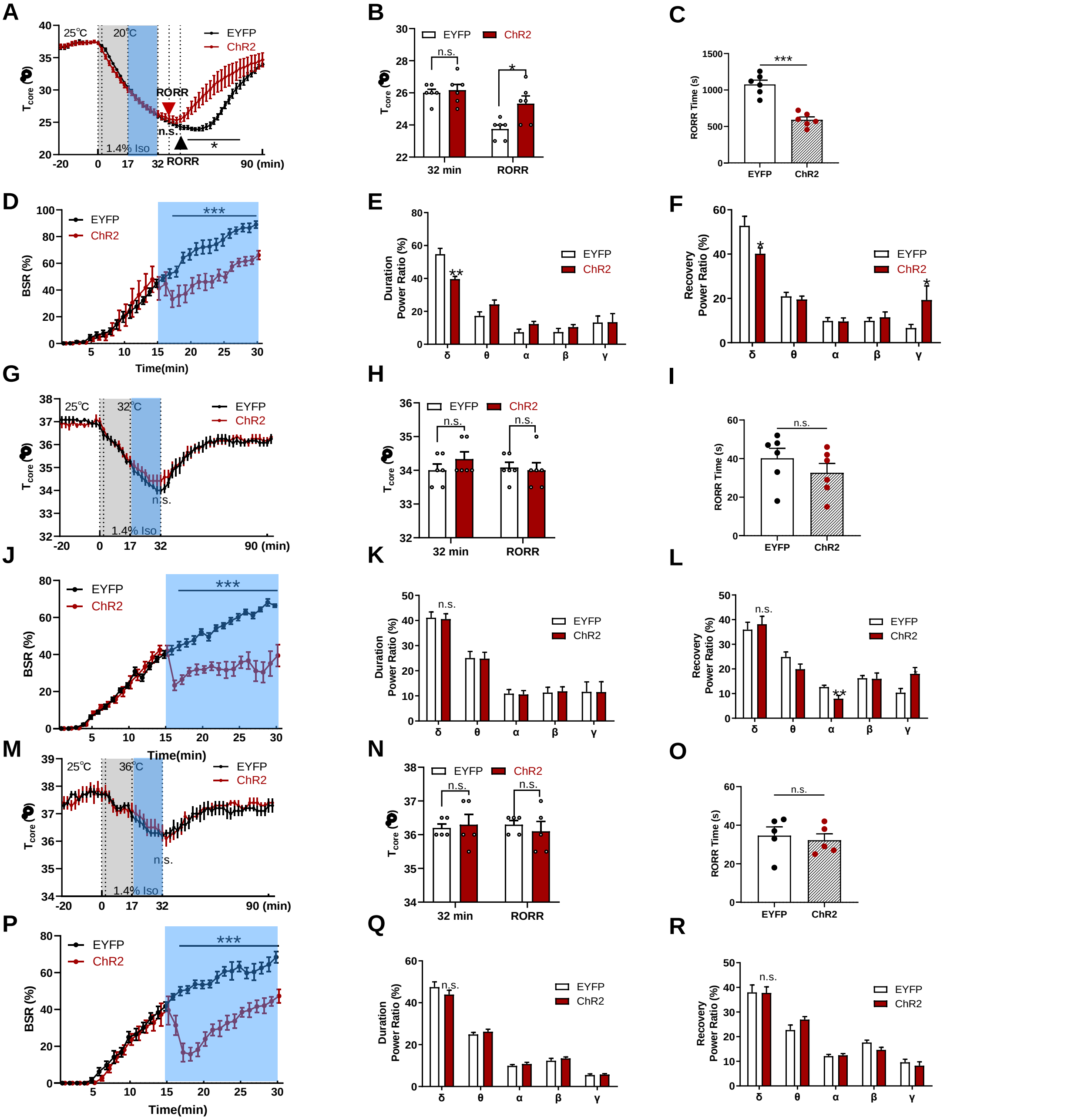

Figure S5. Stage-specific optogenetic activation of DMH^{Vglut2} neurons under different conditions.
(A) T_{core} changes at 20°C of T_a under isoflurane in DMH^{Vglut2} neurons activation mice. (B) The values of T_{core} at different time under isoflurane in DMH^{Vglut2} neurons activation mice. T_{core} at the time of isoflurane cessation (t = 32 min), and at the RORR moment (t_{RORR}). (C) Optogenetic activation of DMH^{Vglut2} neurons shortened the RORR time (591.83 ± 38.39 s *vs*. 1077.0 ± 58.44 s, $p < 0.001$, n = 7). (D) BSR during isoflurane anesthesia procedure (30 min) at 20°C of T_a in DMH^{Vglut2} neurons activation mice. (E) DMH^{Vglut2} neural activation during isoflurane anesthesia procedure (0-30 min) displayed significant decrease in δ power band ($F_{(4,50)} = 13.76$; $p = 0.026$; n = 6). (F) DMH^{Vglut2} neural activation during recovery procedure (from isoflurane ceasing to RORR of mice) displayed significant decrease in δ power band ($F_{(4,50)} = 9.16$; $p = 0.019$; n = 6) and increase in γ power band ($F_{(4,50)} = 9.22$; $p = 0.018$; n = 6). (G) T_{core} changes at 32°C of T_a under isoflurane in DMH^{Vglut2} neurons activation mice. (H) The values of T_{core} at different time under isoflurane in DMH^{Vglut2} neurons activation mice. (I) Optogenetic activation of DMH^{Vglut2} neurons has no influence on the RORR time. (J) BSR during isoflurane anesthesia procedure at 32°C of T_a in DMH^{Vglut2} neurons activation mice. (K) DMH^{Vglut2} neural activation during isoflurane (0-30 min) displayed no significant differences. (L) DMH^{Vglut2} neural activation during recovery procedure (from isoflurane ceasing to RORR of mice) displayed significant decrease in α power band. (M) T_{core} changes at 36°C of T_a under isoflurane in DMH^{Vglut2} neurons activation mice. (N) The values of T_{core} at different time under isoflurane in DMH^{Vglut2} neurons activation mice. (O) Optogenetic activation of DMH^{Vglut2} neurons has no influence on the RORR time. (P) BSR during isoflurane anesthesia procedure at 36°C of T_a in DMH^{Vglut2} neurons activation mice. (Q-R) DMH^{Vglut2} neural activation displayed no significant differences during isoflurane (Q) and recovery procedure (R). All data are presented as mean ± SEM, *$p<0.05$, **$p<0.01$, and ***$p<0.001$, ns, not significant.

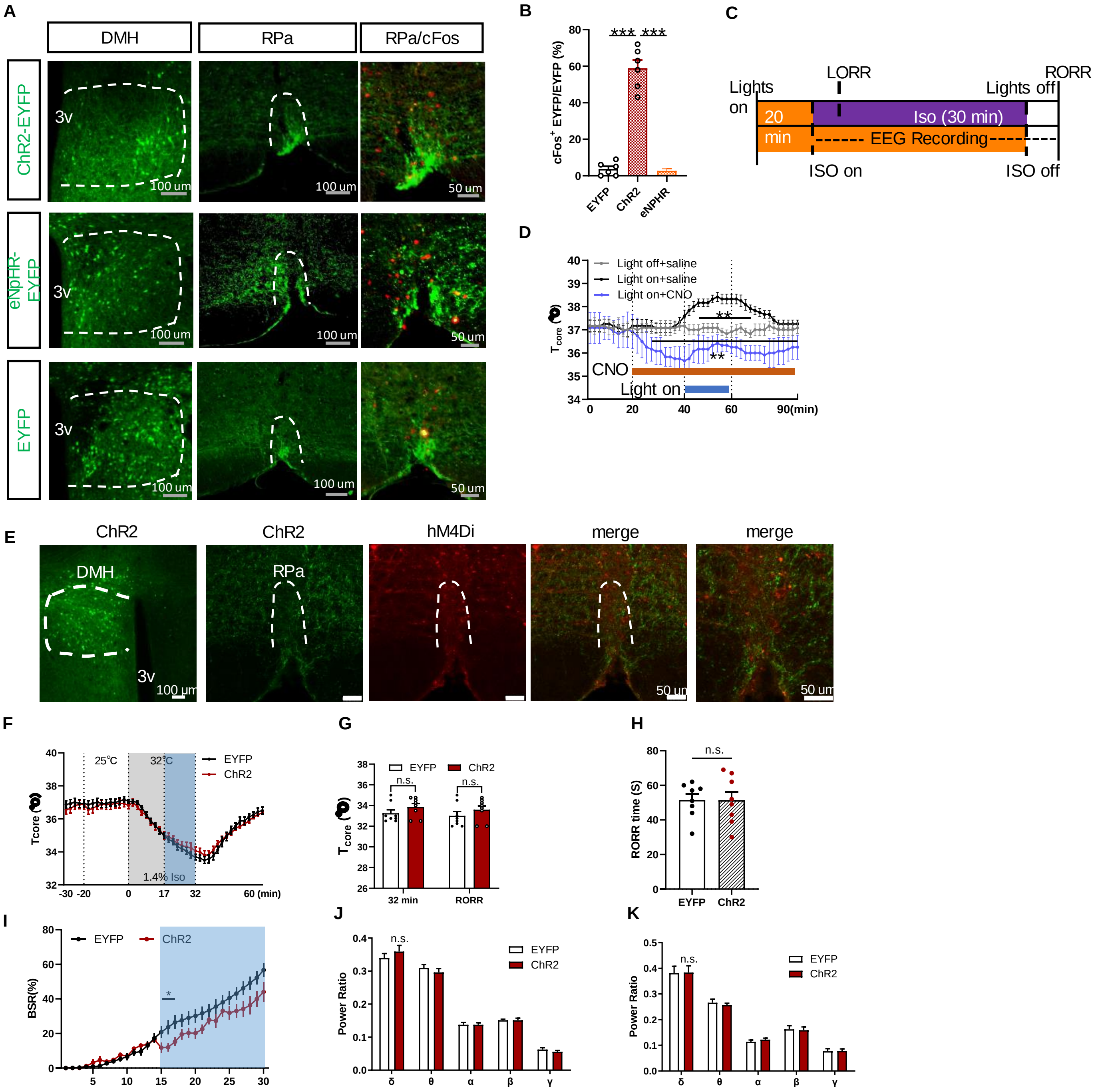

Figure S6. Supplementary data for the validation of the DMH-RPa pathway and optogenetic activation at 32°C T_a

(A) Representative images showing the expression of *ChR2-EYFP* (green) in DMH and RPa region, and c-Fos (red) in the RPa of *Vglut2-ires-Cre* mice after photo-stimulation (30 minutes). Scale bars, 100 μm. (B) Quantification of c-Fos expression in ChR2, eNpHR and EYFP mice. (C) Protocol for the optogenetic inhibition experiment. A core body temperature probe was intraperitoneally injected in the mice two days before the experiment. Yellow shading indicates the optical inhibition phase. (D) T_{core} changes in wake state of mice, photo-stimulation of DMH^{Vglut2}-RPa pathway (t = 40 - 60 min) increased T_{core}, while simultaneously chemogenetic inhibition RPa^{Vglut2} (t = 20 - 90 min) limited the increase of T_{core}. (E) Representative images showing the expression of ChR2 (green) in DMH and hM4Di in RPa region (red), Scale bars, 100 μm and 50 μm. (F) T_{core} changes of ChR2 and EYFP groups under isoflurane at 32°CT_a. (G) The values of T_{core} at different time under isoflurane in ChR2 and EYFP mice. T_{core} at the time of isoflurane cessation (t = 32 min), and at the RORR moment (t_{RORR}). (H) No difference of RORR time in optogenetic activations of the DMH^{Vglut2} to RPa pathway at 32°CT_a. (I) BSR changes of ChR2 and EYFP mice during isoflurane anesthesia procedure. (J-K) Optogenetic activations of the DMH^{Vglut2} to RPa pathway displayed no significant changes in EEG power bands during isoflurane exposure (J), and recovery procedure (K). All data are presented as mean ± SEM, *p<0.05, **p<0.01, and ***p<0.001, n.s., not significant.
